# Supplementary material for: Psoriatic and rheumatoid arthritis joints differ in the composition of CD8+ tissue-resident memory T cell subsets
Source: Cell Rep. 2023 May 16;42(5):112514. doi: 10.1016/j.celrep.2023.112514 (PMC10790246; doi:10.1016/j.celrep.2023.112514)
Supplement: Document S1. Figures S1–S7 [file mmc1.pdf]

Cell Reports, Volume *42*

Supplemental information

Psoriatic and rheumatoid arthritis joints differ in the composition of CD8+ tissue-resident memory T cell subsets

Giovanni A.M. Povoleri, Lucy E. Durham, Elizabeth H. Gray, Sylvine Lalnunhlimi, Shichina Kannambath, Michael J. Pitcher, Pawan Dhami, Thomas Leeuw, Sarah E. Ryan, Kathryn J.A. Steel, Bruce W. Kirkham, and Leonie S. Taams

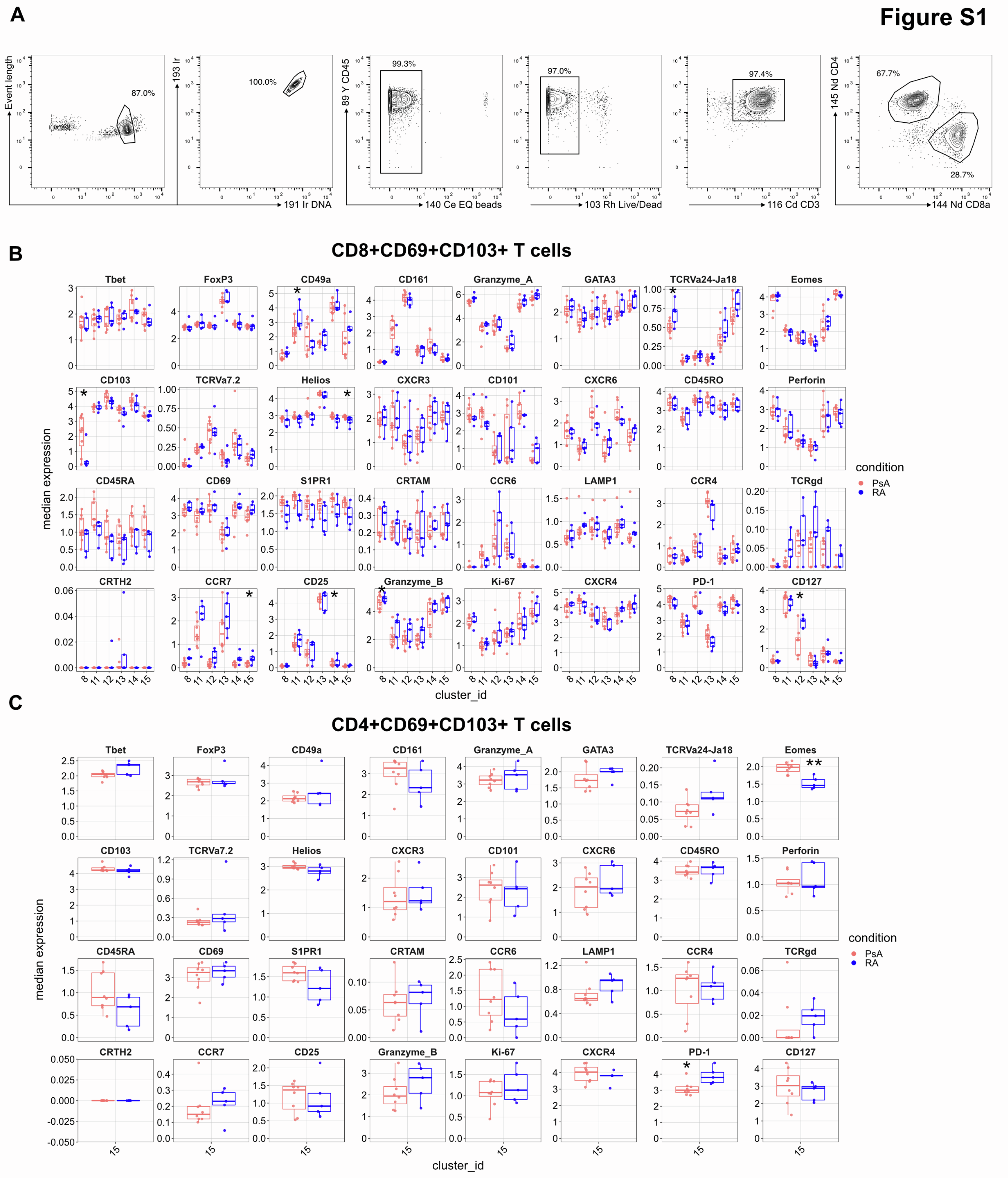

Figure S1. Gating strategy and phenotypic comparison of synovial CD8+CD69+CD103+ and CD4+CD69+CD103+ TRM cells clusters in patients with PsA and RA; Related to Figures 1,2. (A) Representative plots demonstrating the gating strategy for sample cleanup and export of total CD8+ and CD4+ T-cells from patients with PsA or RA pre CyTOF analysis inputted into the modified CATALYST analysis pipeline. The gating strategy is shown for intact cells (191Ir+ DNA stain), cells (191Ir+193Ir+), no beads (89Y CD45+140Ce EQ beads-), live (89Y CD45+ 103Rh-), CD3+ (89Y CD45+ 116Cd CD3+) and CD8+ (144Nd CD8a+ 145Nd CD4-) or CD4+ (144Nd CD8a- 145Nd CD4+) cells. (B,C) Cumulative data showing the median arcsinh-transformed expression of the markers across the identified CD8+CD69+CD103+ (B) or CD4+CD69+CD103+ (C) TRM cell clusters from Figure 1C. Values for the two disease conditions are indicated as PsA in red and RA in blue. Boxplots show median and IQR. Data analysed by multiple Mann-Whitney tests with *p<0.05. Data from patients with PsA (n=8) or RA (n=5) are shown.

Figure S2

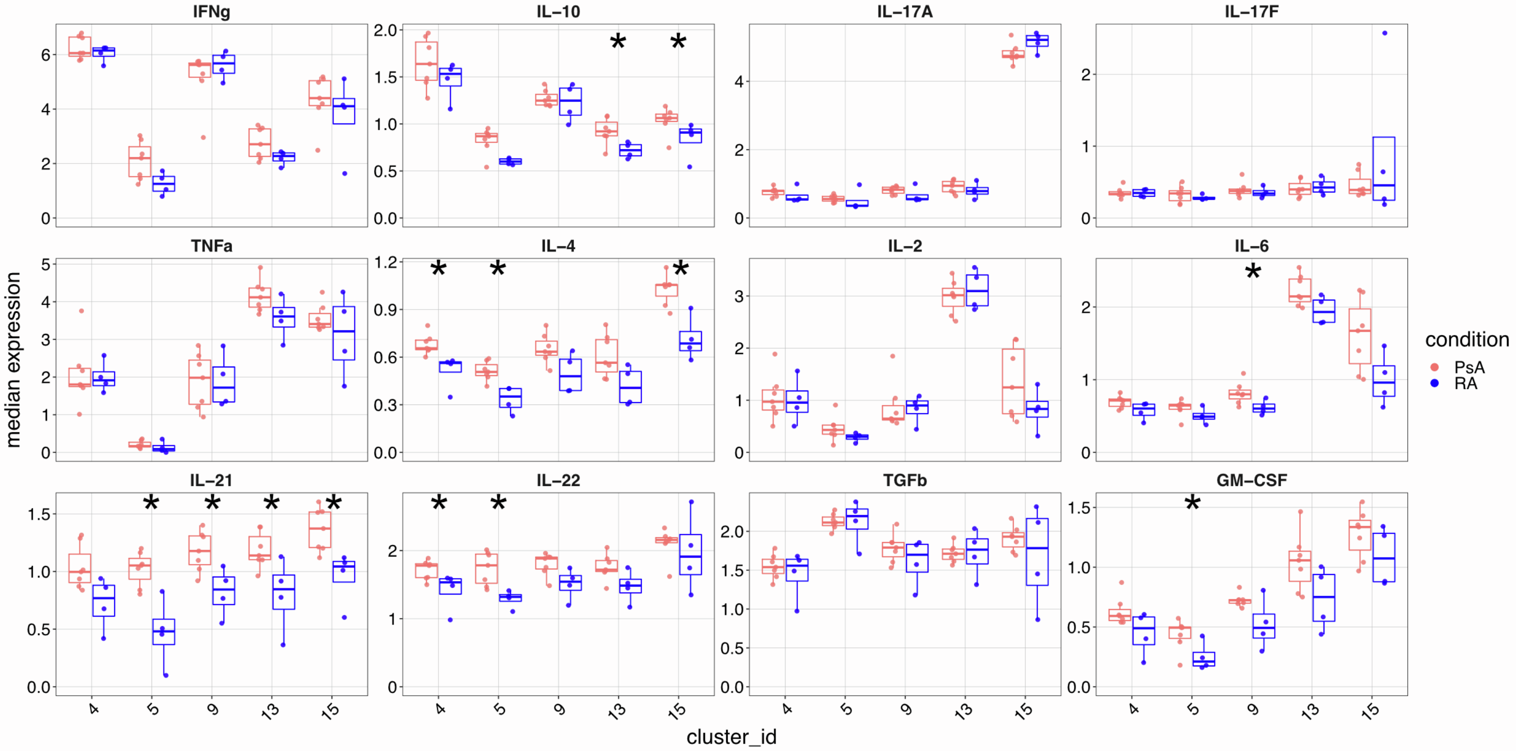

Figure S2. Cytokine expression comparison of synovial CD8+CD69+CD103+ TRM cells clusters in patients with PsA and RA; Related to Figure 3. Cumulative data showing the arcsinh-transformed, median expression of cytokines across the identified CD8+CD69+CD103+ TRM cell clusters from Figure 3B (PsA in red and RA in blue). Boxplots show median and IQR. Data analysed by multiple Mann-Whitney tests and *p<0.05. Data from patients with PsA (n=7) or RA (n=4) are shown.

Figure S3

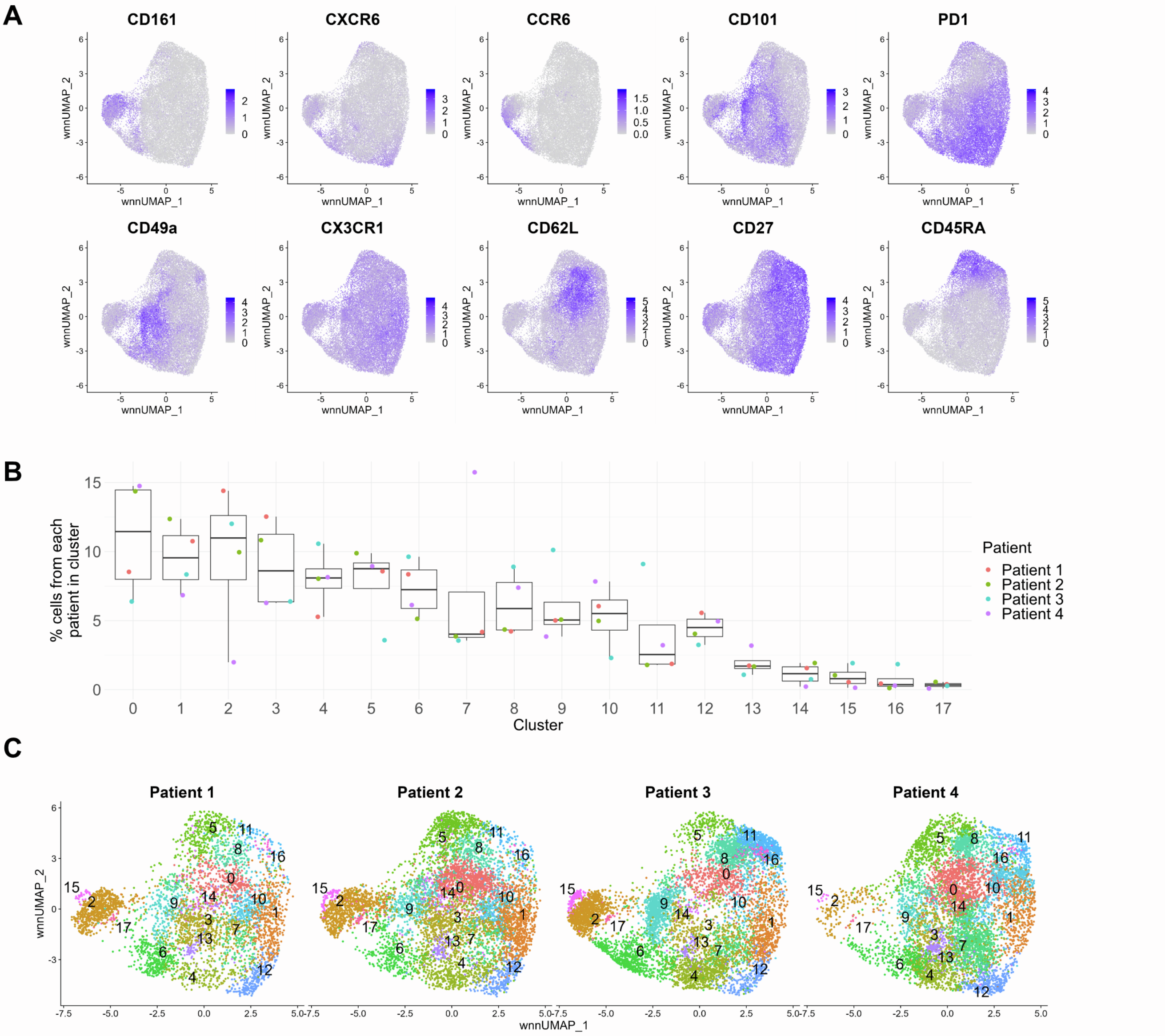

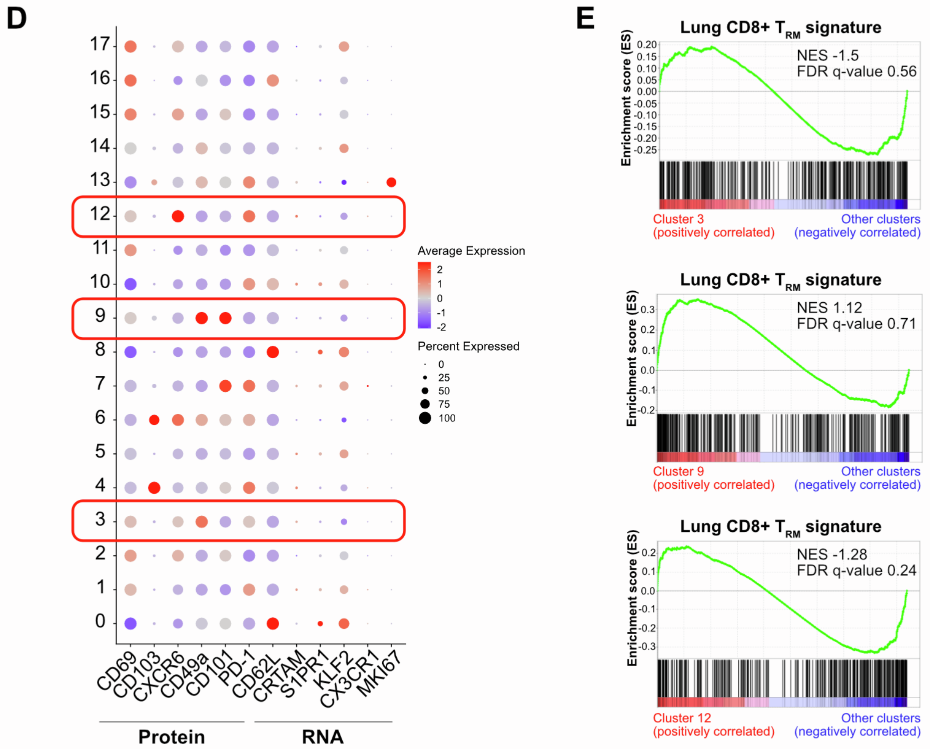

Figure S3. scRNAseq clusters representation among donors and evaluation of synovial CD8+CD69+ CD103- clusters in patients with PsA for TRM cell gene signature; Related to Figure 4. (A) UMAPs showing cell protein expression levels of indicated proteins in CD8+ T-cells from patients with PsA (n=4). (B) Boxplot showing distribution of cells from each patient across clusters (n=4). (C) Individual UMAP for each patient showing cells coloured according to the 18 populations obtained after Seurat clustering. (D) Dot plot heatmap showing the average expression (colour) and percentage of cells (dot size) expressing the indicated genes or proteins associated with a TRM cell phenotype (n=4). (E) GSEA plots for lung CD8+ TRM signature, comparing clusters 3, 9 and 12 with all other clusters; n = 4 per group. Normalized enrichment score (NES) and multiple-test adjusted q-value from GSEA are shown.

Figure S4

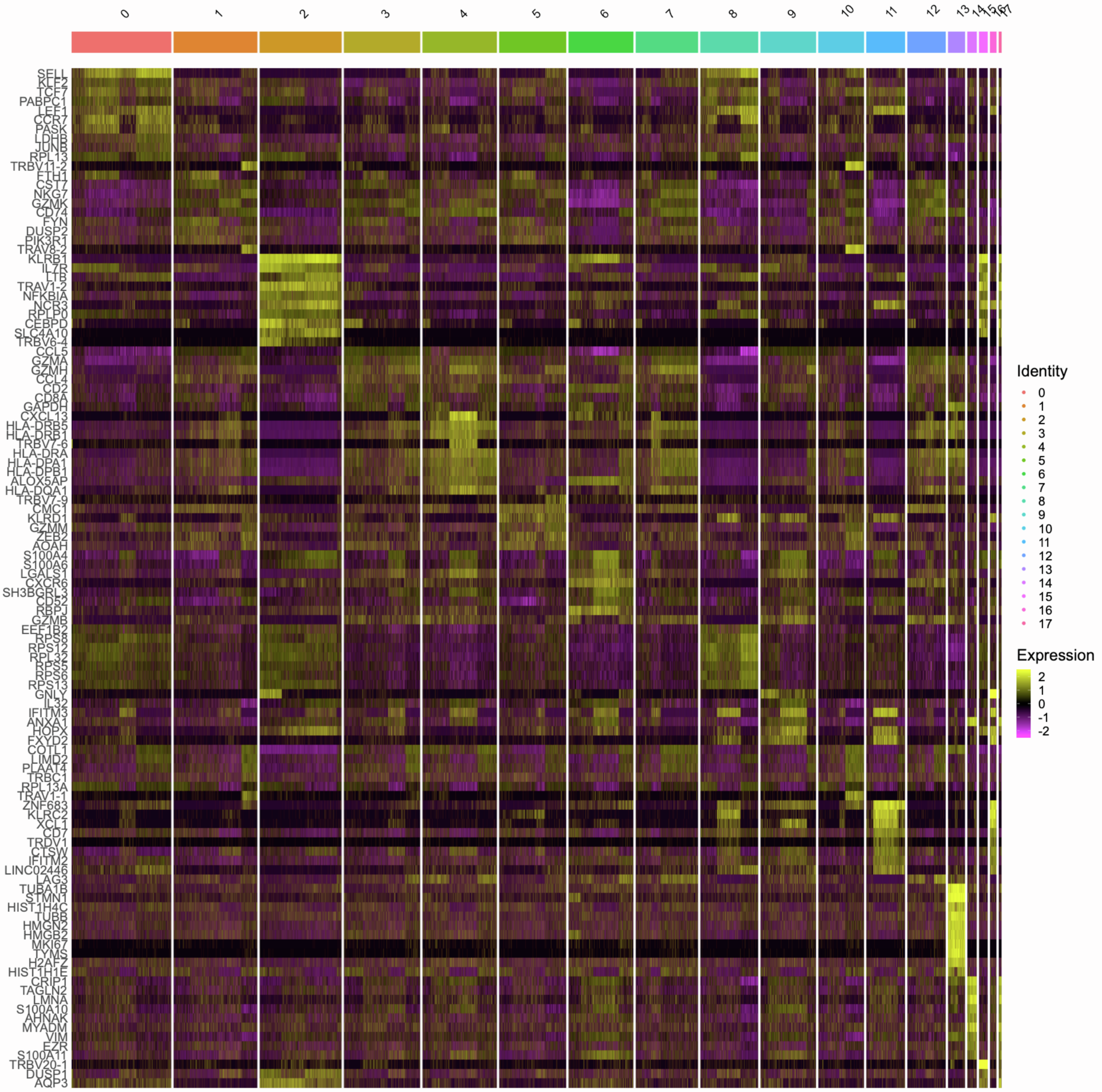

Figure S4. Heatmap of top 10 differentially expressed genes from each cluster identified in synovial CD8+ cells from patients with PsA; Related to Figure 4. Heatmap showing the top 10 upregulated genes for each of the 18 clusters from the integrated analysis of CD8+ T cells from n= 4 patients. Upregulated genes were identified by Wilcoxon rank sum test. Within each cluster, genes are ordered by highest to lowest LogFC of SCTransformed RNA expression. Adjusted P-values < 0.05 were considered statistically significant.

Figure S5

A

Cluster 13

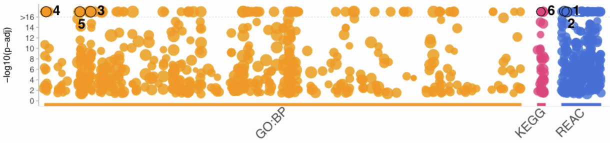

id	term_id	term_name	q_value
1	REAC:R-HSA-1640170	Cell Cycle	1.13E-131
2	REAC:R-HSA-69278	Cell Cycle, Mitotic	6.67E-114
3	GO:0007049	cell cycle	1.16E-114
4	GO:0000278	mitotic cell cycle	1.58E-96
5	GO:0006259	DNA metabolic process	2.98E-72
6	KEGG:04110	Cell cycle	2.83E-25

B

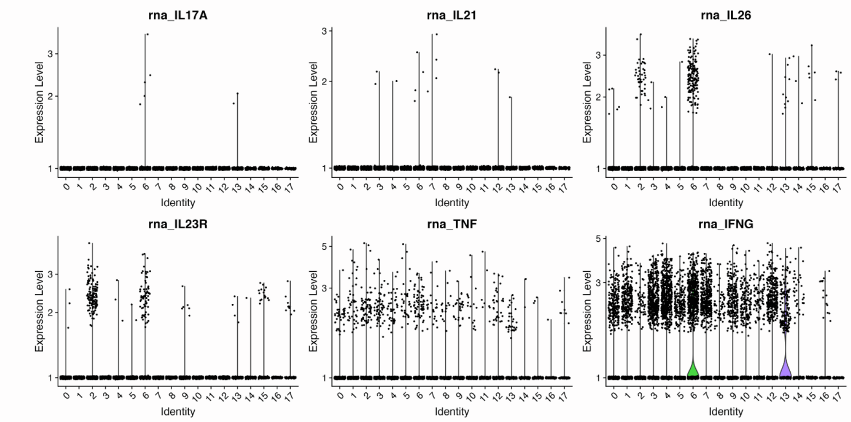

C

Cluster 4

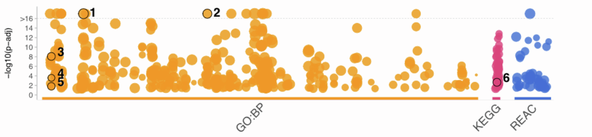

id	term_id	term_name	q_value
1	GO:0006955	immune response	1.10E-31
2	GO:0042110	T cell activation	5.50E-23
3	GO:0001909	leukocyte mediated cytotoxicity	8.54E-09
4	GO:0001913	T cell mediated cytotoxicity	2.6E-04
5	GO:0001910	regulation of leukocyte mediated cytotoxicity	1.5E-02
6	KEGG:04650	Natural killer cell mediated cytotoxicity	2.2E-03

Cluster 6

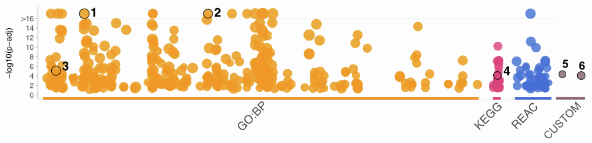

id	term_id	term_name	q_value
1	GO:0006955	immune response	7.19E-19
2	GO:0042110	T cell activation	2.80E-21
3	GO:0002250	adaptive immune response	8.32E-06
4	KEGG:04659	Th17 cell differentiation	8.50E-05
5	Ramesh et al. 2014	Human Th17 signature	2.24E-04
6	Fergusson et al. 2014	CD8+CD161+ and MAIT cells	2.86E-04

D

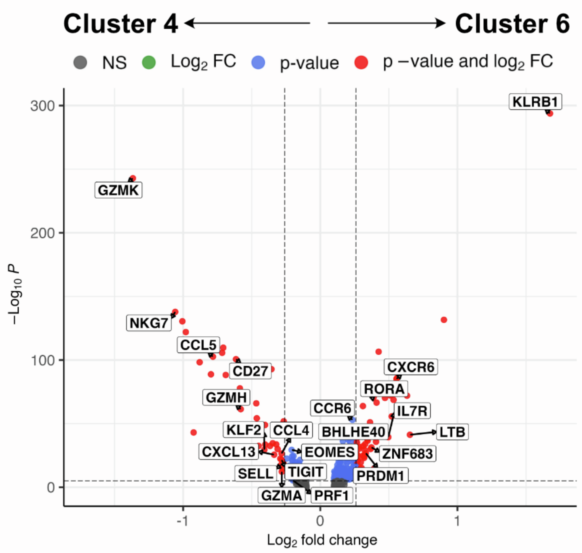

E

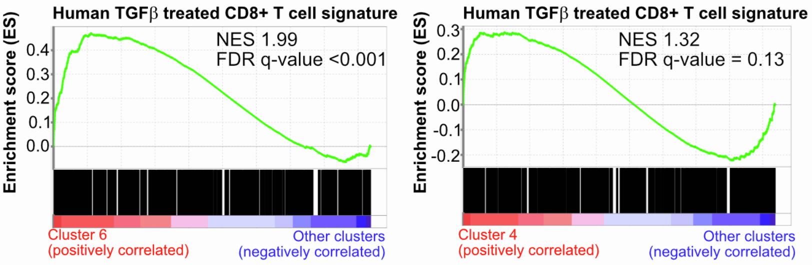

Figure S5. Functional enrichment analysis and gene signature differences between synovial CD8+CD69+CD103+ TRM clusters identified in patients with PsA; Related to Figure 4. (A) Manhattan plot showing functional enrichment analysis of differentially upregulated genes in cluster 13 compared to all other clusters. The x-axis shows the biological functions and pathways data sources (terms) and y-axis shows the enrichment p-values (−log10). Each circle corresponds to a single term from an annotation source (colour) and shows the total number of genes annotated to the corresponding term (circle size). Chosen terms for each cluster are indicated with numbers on the plot and corresponding id, name and statistics are shown in the table below the Manhattan plot. (B) Violin plots showing gene expression levels of type 17 and inflammatory cytokines in the different Seurat clusters identified from SF CD8+ T-cells from patients with PsA (n=4). (C) Manhattan plots, with associated terms and statistics, showing functional enrichment analysis of differentially upregulated genes in clusters 4 (top) and 6 (bottom) compared to all other clusters. (D) Volcano plot showing significant genes differentially expressed in cluster 6 compared to cluster 4. Differentially expressed genes are identified in blue (q-value <0.05) or red (q-value <0.05 with a ±1.2-fold change (n=4 patients). (E) GSEA plots for human TGFβ treated CD8+ T-cell signature, comparing Cluster 6 (left) and 4 (right) with all other clusters; n = 4 per group. Normalized enrichment score (NES) and multiple-test adjusted q-value from GSEA are shown.

Figure S6

A

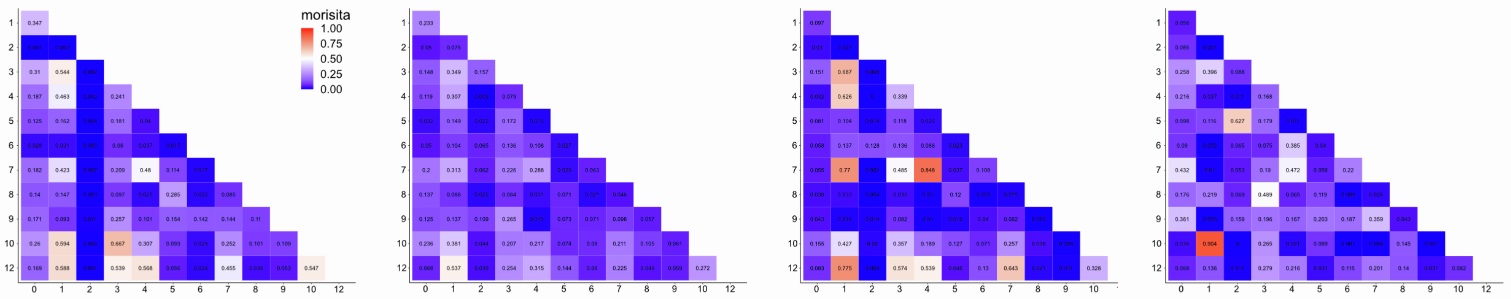

B

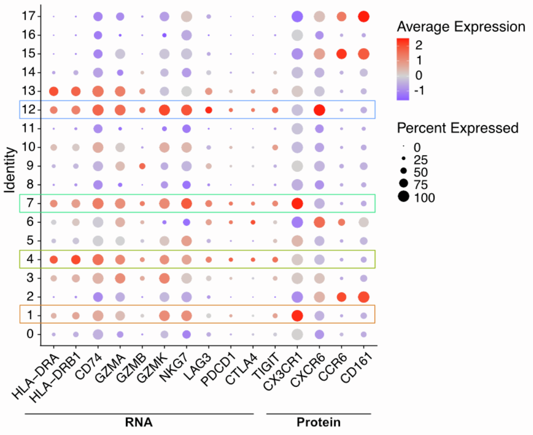

Figure S6. TCR similarity among donors and comparison of gene signatures between clonally similar clusters identified in synovial CD8+ cells from patients with PsA; Related to Figure 5. (A) Heatmaps from each donor of the Morisita similarity index measuring TCR composition overlap between the different clusters; index ranging between 0 as minimal and 1 as maximal similarity. (B) Dot plot showing either protein or gene expression of signature markers that defined clusters from Figure 5D. Dot plot heatmap showing the average expression (colour) and percentage of cells (dot size) expressing the indicated genes or proteins. Clusters 1, 4, 7 and 12 have been highlighted with the corresponding cluster colour for ease of comparison (n=4 patients).

Figure S7

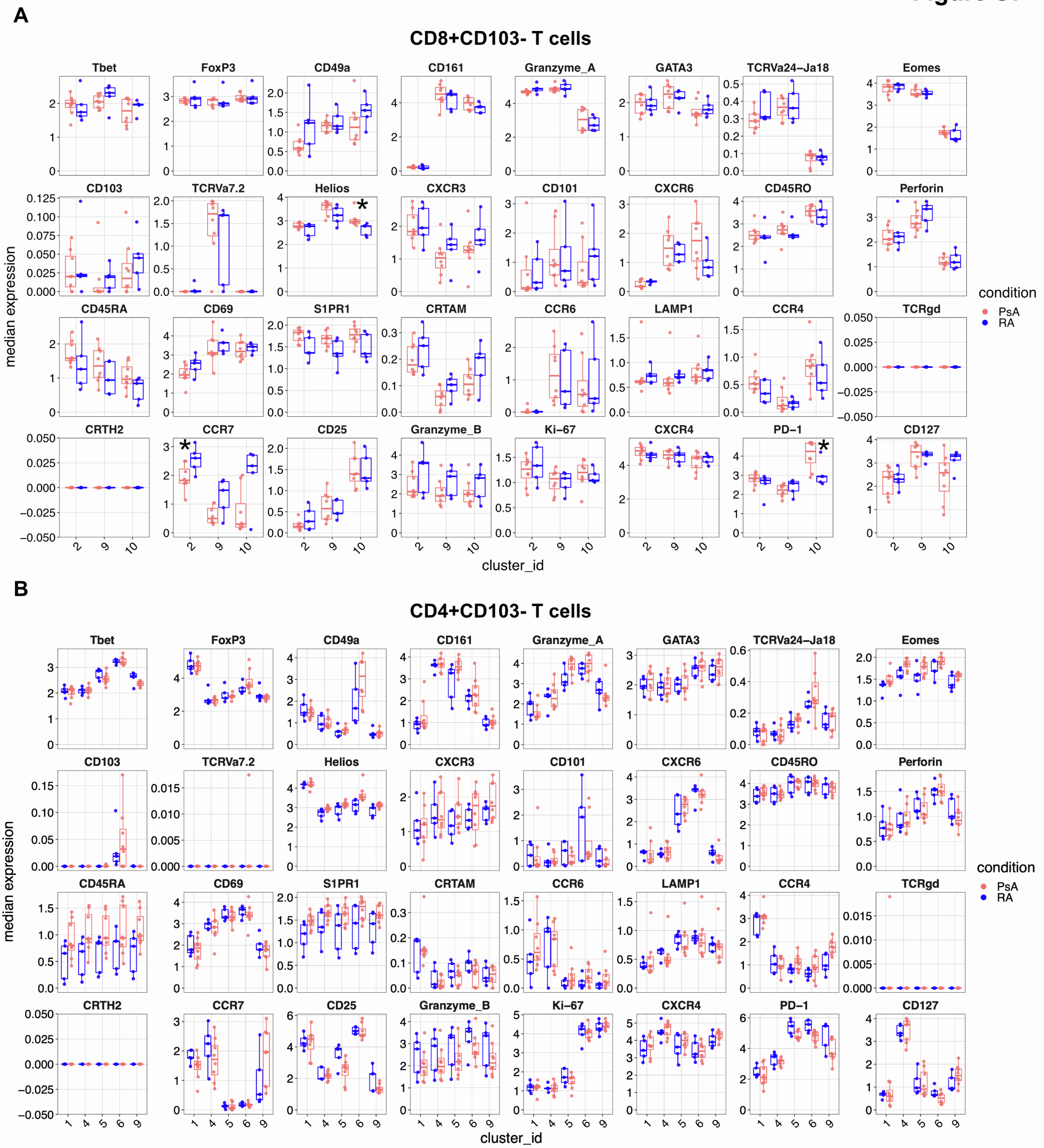

Figure S7. Phenotypic comparison of synovial CD8+CD103- and CD4+CD103- clusters in patients with PsA and RA; Related to Figure 6. Cumulative data showing the median arcsinh-transformed expression of the markers across the significantly enriched CD8+CD103- (A) or CD4+ CD103- cell clusters in either PsA (red) or RA (blue) from Figure 6. Boxplots show median and IQR. Data analysed by multiple Mann-Whitney tests and *p<0.05 . Data from patients with PsA (n=8) or RA (n=5) are shown.
