# Supplementary material for: Psoriatic and rheumatoid arthritis joints differ in the composition of CD8+ tissue-resident memory T cell subsets
Source: Cell Rep. 2023 May 16;42(5):112514. doi: 10.1016/j.celrep.2023.112514 (PMC10790246; doi:10.1016/j.celrep.2023.112514)
Supplement: Document S2. Article plus supplemental information [file mmc5.pdf]

Cell Reports

Article

Psoriatic and rheumatoid arthritis joints differ in the composition of CD8+ tissue-resident memory T cell subsets

Graphical abstract

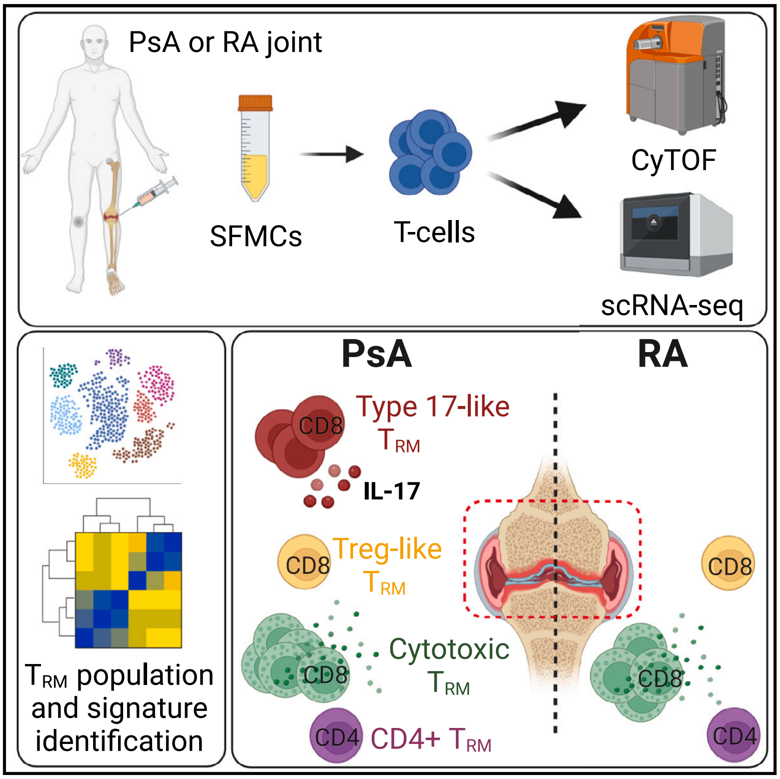

Authors

Giovanni A.M. Povoleri, Lucy E. Durham, Elizabeth H. Gray, ..., Kathryn J.A. Steel, Bruce W. Kirkham, Leonie S. Taams

Correspondence

leonie.taams@kcl.ac.uk

In brief

Combining CyTOF and scRNA-seq immunophenotyping, Povoleri et al. reveal quantitative differences in synovial CD8+ tissue-resident memory T (T_{RM}) cell subset composition between psoriatic and rheumatoid arthritis. The proinflammatory type 17 profile enriched in T_{RM} cells from psoriatic arthritis joints may potentially explain the differential responses to IL-17 blockade between the diseases.

Highlights

- Human arthritic joints contain distinct subsets of resident memory T (T_{RM}) cells
- Psoriatic and rheumatoid arthritis joints differ in T_{RM} subset composition
- Psoriatic arthritis is enriched for pro-inflammatory type 17 T_{RM} and other T cells
- Rheumatoid arthritis is enriched for T cells with a cytotoxic profile

Povoleri et al., 2023, Cell Reports *42*, 112514
May 30, 2023 © 2023 The Author(s).
https://doi.org/10.1016/j.celrep.2023.112514

CellPress

Cell Reports

CellPress
OPEN ACCESS

Article

Psoriatic and rheumatoid arthritis joints differ in the composition of CD8+ tissue-resident memory T cell subsets

Giovanni A.M. Povoleri,[1,6] Lucy E. Durham,[1,6] Elizabeth H. Gray,[1,6] Sylvine Lalnunhlimi,[1] Shichina Kannambath,[2] Michael J. Pitcher,[3] Pawan Dhami,[2] Thomas Leeuw,[4] Sarah E. Ryan,[1] Kathryn J.A. Steel,[1] Bruce W. Kirkham,[5] and Leonie S. Taams[1,7,*]

[1]Centre for Inflammation Biology and Cancer Immunology (CIBCI), Department of Inflammation Biology, School of Immunology & Microbial Sciences, King's College London, London SE1 1UL, UK

[2]BRC Genomics Core, NIHR Biomedical Research Center, Guy's and St Thomas' NHS Foundation Trust and King's College London, Guy's Hospital, London SE1 9RT, UK

[3]Peter Gorer Department of Immunobiology, School of Immunology and Microbial Sciences, King's College London, London SE1 9RT, UK

[4]Immunology & Inflammation Research TA, Sanofi-Aventis Deutschland GmbH, Industriepark Hoechst, 65926 Frankfurt am Main, Germany

[5]Rheumatology Department, Guy's Hospital, Great Maze Pond, London SE1 9RT, UK

[6]These authors contributed equally

[7]Lead contact

*Correspondence: leonie.taams@kcl.ac.uk

https://doi.org/10.1016/j.celrep.2023.112514

SUMMARY

CD69+CD103+ tissue-resident memory T (T_{RM}) cells are important drivers of inflammation. To decipher their role in inflammatory arthritis, we apply single-cell, high-dimensional profiling to T cells from the joints of patients with psoriatic arthritis (PsA) or rheumatoid arthritis (RA). We identify three groups of synovial CD8+CD69+CD103+ T_{RM} cells: cytotoxic and regulatory T (Treg)-like T_{RM} cells are present in both PsA and RA, while CD161+CCR6+ type 17-like T_{RM} cells with a pro-inflammatory cytokine profile (IL-17A+TNFα+ IFNγ+) are specifically enriched in PsA. In contrast, only one population of CD4+CD69+CD103+ T_{RM} cells is detected and at similarly low frequencies in both diseases. Type 17-like CD8+ T_{RM} cells have a distinct transcriptomic signature and a polyclonal, but distinct, TCR repertoire. Type 17-like cells are also enriched in CD8+CD103− T cells in PsA compared with RA. These findings illustrate differences in the immunopathology of PsA and RA, with a particular enrichment for type 17 CD8+ T cells in the PsA joint.

INTRODUCTION

Psoriatic arthritis (PsA) and rheumatoid arthritis (RA) are the most common types of inflammatory arthritis, and they frequently cause significant disability and reduction in quality of life.[1] Inflammation of the synovial joints is a key characteristic of both diseases; however, PsA and RA have marked differences in serology and genetic associations and show different responses to targeted therapies.[1] Despite major improvements in effective therapies, both diseases run a chronic course with recurrent disease flares and low frequencies of drug-free prolonged remission states. It is not known why inflammation specifically affects the joints or persists despite current therapies suppressing inflammatory pathways. Critically, there are no current treatments that specifically target cells that reside only in the inflamed tissue. Improved knowledge of the cells or factors that drive chronic joint inflammation would create a novel basis for moving toward joint targeted treatments to stop development or progression of arthritis by inhibiting pathways that drive persistent disease.

The recent identification of a functional T cell subset, called tissue-resident memory T cells (T_{RM} cells), may provide key insights into mechanisms driving chronic immune-mediated inflammatory diseases.[2–4] T_{RM} cells, typically identified by expression of CD69 and/or CD103, are a specialized subset of effector T cells that provide tissue-specific immune surveillance.[2,5,6] Most T_{RM} cells remain permanently in the tissue and do not recirculate in the blood. However, recent studies have shown that a small proportion of T_{RM} cells can exit the tissues and persist in the blood, seed in distant tissues or lymph nodes, or *trans*-differentiate into other memory subsets.[7–10] While most previous studies have focused on CD8+ T_{RM} cells, these cells exist in both the CD4+ and CD8+ T cell compartment.[11–13] They are mainly located in epithelial tissues such as skin, lung, and gut, where it is proposed they protect against infection and cancer but may also contribute to chronic inflammation.[11,12,14–17] Accumulating evidence indicates that T_{RM} cells

This is an open access article under the CC BY license (http://creativecommons.org/licenses/by/4.0/).

Table 1. Demographic and clinical parameters of patients with PsA and RA included for CyTOF and scRNA-seq

	Patients included for CyTOF		Patients included for scRNA-seq
	PsA (n = 7)	RA (n = 5)	PsA
Male/female	4/3	1/4	2/2
Age (years, mean ± SD)	42 ± 15	57 ± 14	36 ± 9.7
Disease duration (years, mean ± SD)	11 ± 8.3	7.7 ± 4.2	8.6 ± 7.1
DAS28	6.4 ± 1.3 (n = 2)	4.7 ± 1.3 (n = 5)	5.4 ± 0 (n = 1)
Rheumatoid factor/ACPA+ (%)	0	60	0
Medication	nil (n = 1); NSAIDs (n = 1); Pred (n = 3); MTX (n = 2); MTX/naproxen (n = 1)	nil (n = 2); MTX/Pred (n = 1); SSZ (n = 2)	nil (n = 1); Pred (n = 1); MTX (n = 2)

No significant bias or association due to patient clinical parameters, sex/gender, age, disease duration, or treatment strategy was observed in the data analysis. ACPA, anti-citrullinated protein antibody; DAS28, disease activity score in 28 joints; MTX, methotrexate; NSAIDs, non-steroidal anti-inflammatory drugs; Pred, prednisolone; SD, standard deviation; SSZ, sulfasalazine.

are present in the inflamed joints of patients with PsA, RA, and juvenile idiopathic arthritis, where they may drive chronic inflammation and disease flares.[18–22]

In humans, T_{RM} cells share a core transcriptional signature including upregulation of extracellular molecules that facilitate tissue retention (CD103, CXCR6, CD49a, and CRTAM), inhibitory receptors (PD-1 and CTLA4), and cytokines (IFNγ, IL-2, IL-17A, and IL-10), downregulation of extracellular molecules required for tissue egress (CD62L and S1PR1), and low basal proliferation.[15] Despite this shared signature, T_{RM} cells are heterogeneous and can differ both within and between tissues. In human skin, for instance, CD49a expression differentiates two functionally distinct T_{RM} cells: cytotoxic or IL-17-producing.[14] Furthermore, the conditions required for the differentiation and maintenance of T_{RM} cells can differ between tissues, as shown by evidence that transforming growth factor β (TGFβ) signaling is required for murine skin but not liver T_{RM} cells.[23]

Here, we investigated the hypothesis that T_{RM} cells are present in the inflamed joints of patients with inflammatory arthritis but that these cells are numerically, phenotypically, and/or functionally different between PsA and RA. Using high-dimensional profiling (CyTOF and single-cell RNA sequencing [scRNA-seq]), we performed quantitative and qualitative analysis of synovial fluid CD8+ and CD4+ CD69+CD103+ T_{RM} cells from patients with PsA or RA. We identified one population of synovial CD4+CD69+CD103+ T_{RM} cells, which were present at similarly low frequencies in both PsA and RA. In contrast, multiple distinct populations of CD8+CD69+CD103+ T_{RM} cells were found enriched in PsA. In particular, an enrichment of type 17-like cells was found among CD8+CD103+ T_{RM} cells as well as among CD8+CD103− T cells in PsA compared with RA. We show that type 17 CD8+CD69+CD103+ T_{RM} cells in PsA have a pro-inflammatory cytokine profile and a distinct transcriptomic signature. Furthermore, clonality analysis revealed a polyclonal but distinct T cell receptor (TCR) repertoire for synovial type 17-like CD8+CD69+CD103+ T_{RM} cells compared with other T_{RM} and CD8+CD103− T cells in patients with PsA. These findings add substantively to the accumulating evidence that the immunopathologies of PsA and RA are different, with a particular role for type 17-like CD8+CD103+ T_{RM} and CD8+CD103− T cells in PsA.

RESULTS

Multiple populations of CD8+CD69+CD103+ T_{RM} cells are enriched in the synovial fluid of patients with PsA compared with RA

We first sought to determine whether there were differences in joint-derived CD8+ or CD4+ T_{RM} cells between PsA (n = 8 samples from n = 7 different patients) versus RA (n = 5 samples from n = 5 patients) (see Table 1 for demographic and clinical information). For this, we performed mass cytometry (CyTOF) on CD3+ T cells isolated from cryopreserved synovial fluid mononuclear cells (SFMCs) and measured the expression of multiple chemokine receptors, transcription factors, T cell receptors, and effector and cytotoxic molecules on exported CD8+ and CD4+ T cells (CyTOF panel I; key resources table). We focused our analysis on live T cells using a customized version of the CATALYST pipeline[24] (Figure S1A). We first applied multidimensional scaling (MDS) using median marker expression across all cells to evaluate differences between the patient samples. This analysis did not reveal disease-specific clustering of synovial CD8+ or CD4+ T cells, suggesting a degree of phenotypic similarity between the two patient cohorts (Figure 1A). Next, we generated uniform manifold approximation and projections (UMAPs) of the expression of CD69 and CD103, canonical markers of human T_{RM} cells, on synovial T cells from patients with PsA or RA. CD103 expression in synovial CD8+ T cells, and to a lesser degree CD4+ T cells, clearly clustered T_{RM} cells in both PsA and RA, with a higher abundance of CD103+ cells within the CD8+ T cell compartment (Figure 1B). The data also suggested higher expression of CD103 in patients with PsA compared with RA (Figure 1B). In contrast, we found that the majority of synovial CD8+ and CD4+ T cells expressed high levels of CD69, which most likely reflects the state of T cell activation in this inflammatory environment rather than identifying those cells as CD69+CD103− T_{RM} cells. Hence, in this study we defined T_{RM} cells as CD69+CD103+ double-positive cells.

Cell population identification was then conducted by unsupervised clustering using the FlowSOM and ConsensusClusterPlus packages, which resolved both CD8+ and CD4+ T cells into 15 clusters across all patient samples, with each population characterized by a different expression profile (Figure 1C). These 15

CellPress
OPEN ACCESS

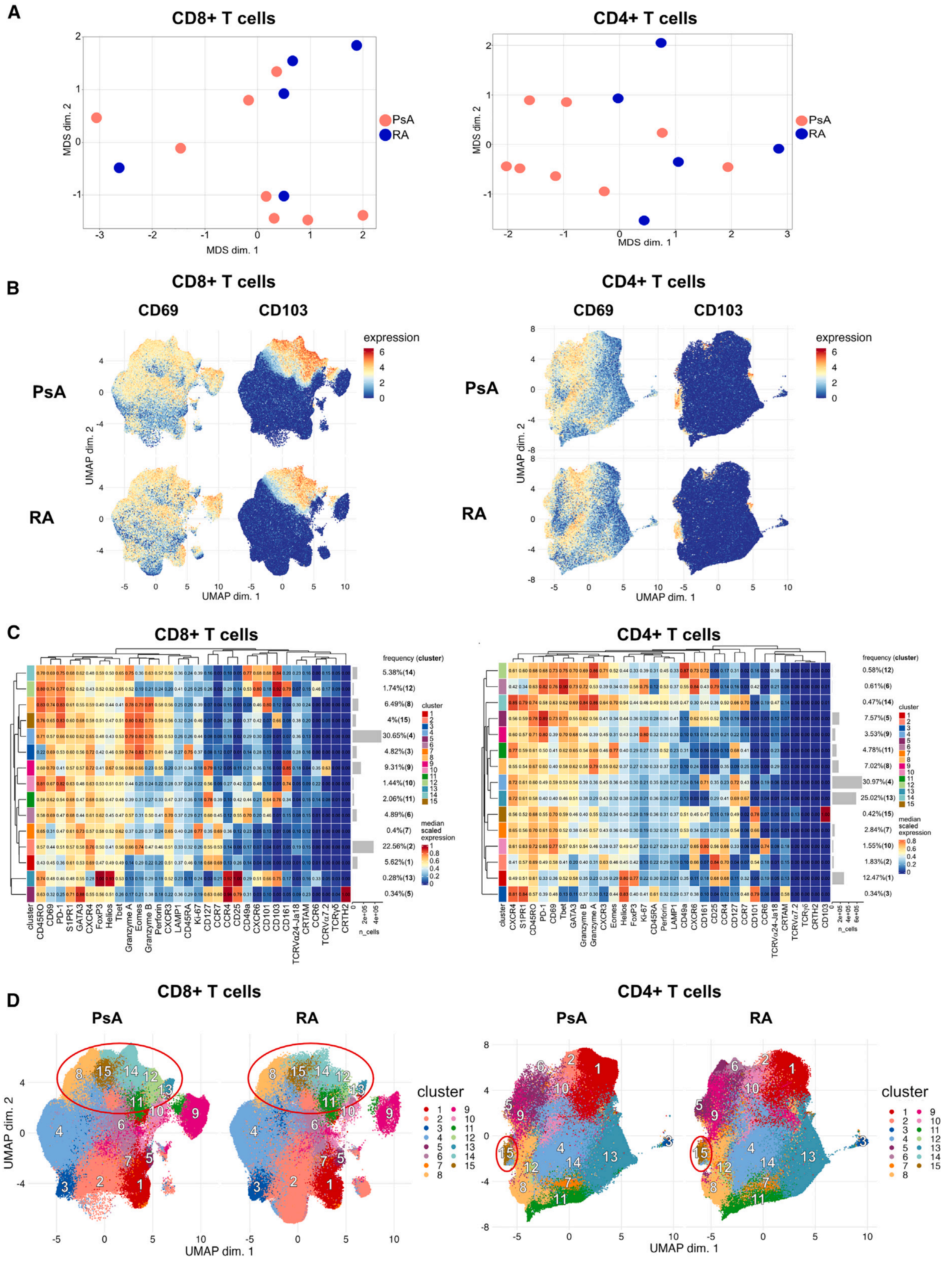

(legend on next page)

clusters were mapped onto UMAPs of CD8+ and CD4+ T cells from patients with PsA or RA for direct comparison (Figure 1D). Based on the co-expression of CD69 and CD103, FlowSOM analysis identified clusters 8, 11, 12, 13, 14, and 15 as T_{RM} cells within CD8+ T cells and cluster 15 as T_{RM} cells within CD4+ T cells (Figure 1C). Of note, cluster 8 showed an overall lower expression of the T_{RM} marker CD103, which was due to its expression being limited to a subset of cells in that cluster. However, FlowSOM was not able to separate out a distinct cluster due to the shared phenotypic characteristics of the CD103+ and CD103− cells within this cluster.

We investigated if there were quantitative differences in these CD8+ and CD4+ T_{RM} cell clusters between PsA and RA. Frequency analysis revealed that the different CD8+ T_{RM} cell populations all appeared enriched in PsA compared with RA, with a statistically significant increase in PsA for cluster 12, which was virtually absent in patients with RA (Figure 2A). In contrast, CD4+ T_{RM} cell frequencies were similarly low in both PsA and RA (Figure 2B).

To identify a signature for these clusters, we performed marker enrichment modeling (MEM) analysis[25] to quantify subset-specific, positive and negative marker enrichment. MEM scores confirmed high positive CD103 enrichment for all CD8+ and CD4+ T_{RM} clusters, which served as positive control for the classification of these subsets as T_{RM} cells (Figure 2C). This MEM analysis revealed several major CD8+ T_{RM} phenotypes: clusters 8, 14, and 15 shared a positive enrichment for cytotoxic mediators perforin and granzymes A and B together with enrichment for CXCR6 and the transcription factor Eomes, indicating a cytotoxic effector profile; cluster 11 was characterized by enrichment for CD127, CD101, and CCR7; cluster 12 showed CXCR6 with CD161 and CCR6 enrichment, suggesting a type 17-like T_{RM} profile; finally, cluster 13 was distinguished by CXCR6 with CD25, Foxp3, Helios, and CD101, but also CD161 and CCR6, enrichment, indicating a regulatory T (Treg)-like T_{RM} profile with a type 17 signature (Figure 2C). MEM analysis of CD4+ T_{RM} cells revealed a type 17-like T_{RM} profile with CD161, CD127, and CXCR6 enrichment (Figure 2D). CD49a, a marker previously associated with CD8+ T_{RM} cells poised for cytotoxic function in human skin,[14,26] was expressed at varying degrees in several clusters (Figures 2C and 2D).

While the frequencies of the CD8+ T_{RM} cell subsets were different between PsA and RA (Figure 2A), there were only minor phenotypic differences when comparing these populations between the two diseases (Figure S1B). A similar result was seen for CD4+ T_{RM} cells (Figure S1C). This indicates that the T_{RM} cell subsets differ quantitatively, at least in the CD8 compartment, rather than qualitatively between PsA and RA.

Cytokine expression profiles of synovial CD8+CD69+CD103+ T_{RM} cell populations

To functionally characterize the different CD8+ T_{RM} populations, we performed CyTOF on synovial CD3+ T cells that were stimulated for 3 h with PMA/ionomycin in the presence of GolgiStop followed by staining with a modified cytokine focused panel (CyTOF panel II; key resources table). As expected, PMA/ionomycin greatly increased the expression of CD69 in most of the cells; however, a clear clustering of CD103-expressing cells could still be found and, concordant with our previous results, higher CD103 expression and increased frequency of CD103+ cells were observed in synovial CD8+ T cells from patients with PsA compared with RA (Figure 3A). FlowSOM resolved the synovial CD8+ T cells into 15 clusters (Figure 3B and 3C), with clusters 4, 5, 9, 13, and 15 identified as T_{RM} cells based on their co-expression of CD103 and CD69 (Figure 3B). Of these, clusters 9, 13, and 15 were all highly significantly enriched in synovial CD8+ T cells from patients with PsA compared with RA (Figure 3D). MEM analysis of the CD8+ T_{RM} clusters revealed distinct cytokine signatures: clusters 9, 13, and 15 were all characterized by CD161 enrichment, associating them to a type 17-like T_{RM} profile. These clusters showed polyfunctional but different cytokine production profiles with cluster 9 enriched for IFNγ and TNFα, cluster 13 for TNFα and IL-2, and cluster 15 showing IL-17A, IFNγ, and TNFα as well as CXCR6 enrichment (Figures 3E and S2). Cluster 4 showed IFNγ enrichment together with Eomes, which correlated it to the cytotoxic effector profile from panel I, while cluster 5 showed CD25, Foxp3, and Helios enrichment but no positive cytokine enrichment, correlating it to a Treg-like profile. These data suggest that the different CD8+ T_{RM} cell populations are characterized by discrete cytokine profiles. The expression of other cytokines tested (IL-4, IL-6, IL-10, IL-17F, IL-21, IL-22, GM-CSF, and TGFβ) was relatively low in these five T_{RM} clusters, although some PsA T_{RM} clusters showed higher expression levels compared with their RA counterparts (Figure S2).

scRNA-seq analysis defines two distinct signatures for synovial CD8+CD69+CD103+ T_{RM} cells in patients with PsA

Our analysis thus far revealed that multiple populations of CD8+CD69+CD103+ T_{RM} cells are enriched in the SF of patients

Figure 1. Mass cytometry identifies distinct populations of synovial CD8+CD69+CD103+ and CD4+CD69+CD103+ T_{RM} cells in patients with PsA and RA

Synovial fluid CD3+ T cells from patients with PsA (n = 8) or RA (n = 5) were stained with panel I (without stimulation); live CD8+ and CD4+ T cells were gated and analyzed independently; data from CD8+ T cells (left panels) and CD4+ T cells (right panels) are shown.

(A) MDS plot for PsA (red) and RA (blue) samples; clustering was based on all marker median expression.

(B) UMAPs based on the arcsinh-transformed expression of 32 markers evaluated in total CD8+ (left) and CD4+ (right) T cells. From each sample, 60,000 cells were randomly selected. UMAPs show cell expression levels of CD69 (left column) and CD103 (right column), stratified by disease: PsA (top row) and RA (bottom row). Cells were clustered based on surface and intracellular markers.

(C) Heatmap of the median marker intensities of the 32 markers across the 15 populations obtained with FlowSOM. Color and value in the heatmap represent the median arcsinh-transformed, 0- to 1-scaled marker expression calculated over cells from all samples; hierarchical similarity between the 15 clusters calculated by Euclidean distance with average linkage. Barplots alongside the rows and values show the relative cluster sizes and ID.

(D) UMAPs generated as in (B) stratified by disease and showing cells colored according to the 15 populations obtained with FlowSOM and characterized in (C); red circles highlight T_{RM} cell clusters.

CellPress
OPEN ACCESS

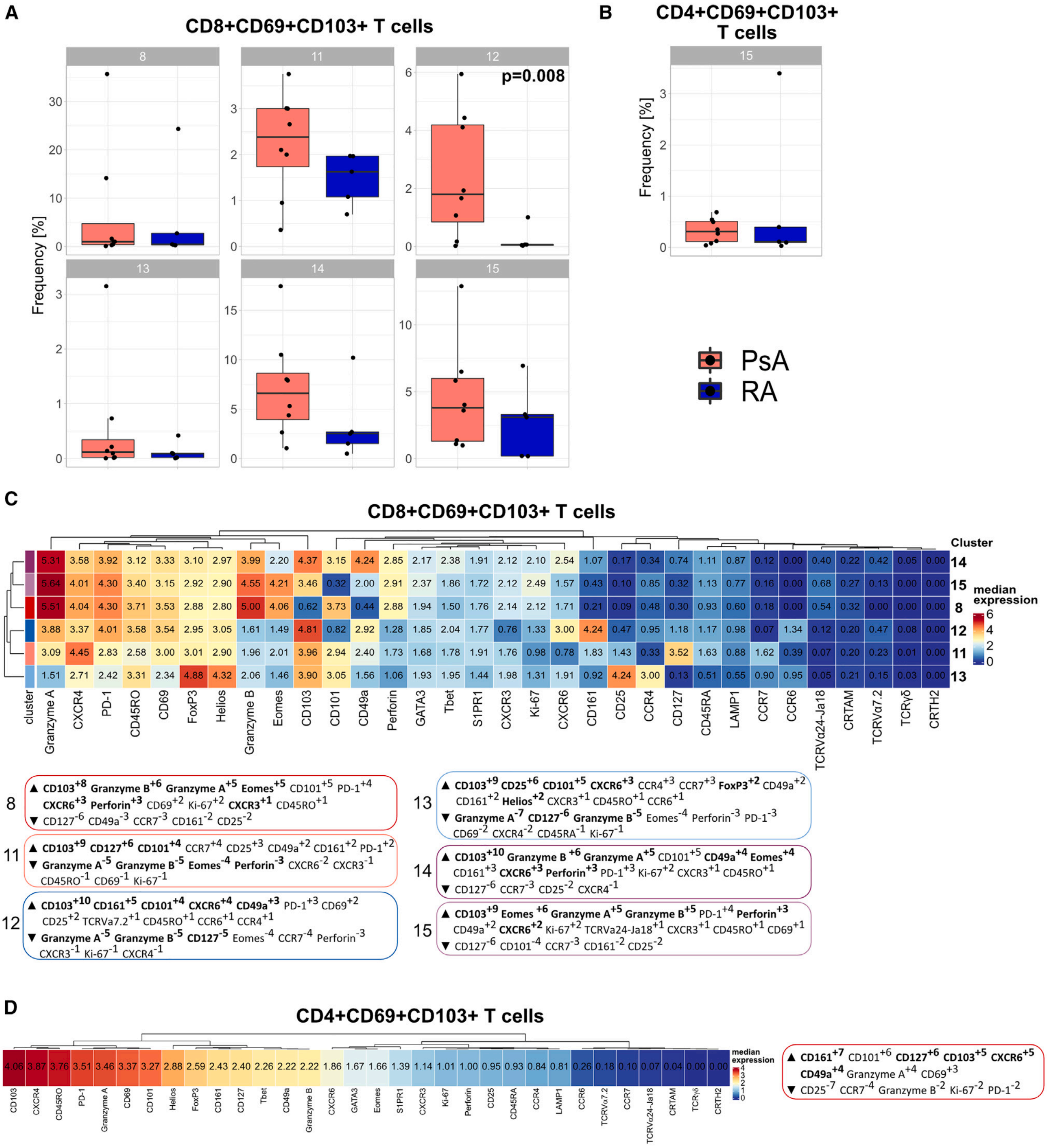

Figure 2. Multiple populations of synovial CD8+CD69+CD103+ T_{RM} cells are enriched in patients with PsA compared with RA

(A and B) Cumulative data showing the relative abundance of CD8+CD69+CD103+ (A) and CD4+CD69+CD103+ (B) T_{RM} cell clusters from Figure 1C, identified by co-expression of CD69 and CD103, in patients with PsA (red) and RA (blue); boxplots show median ± interquartile range (IQR); PsA (n = 8) and RA (n = 5) data analyzed by generalized linear mixed models (GLMMs) and p <0.05 reported.

(C and D) Heatmap of the median marker intensities of the 32 markers across the 6 CD8+CD69+CD103+ T_{RM} cell populations (C) and 1 CD4+CD69+CD103+ T_{RM} cell population (D). Heatmap details as in Figure 1. MEM labels (boxed) were computationally generated for each of the CD8+CD69+CD103+ T_{RM} cell populations (C) and the CD4+CD69+CD103+ T_{RM} cell population (D), and markers were assigned positive (up arrow) or negative (down arrow) enrichment values within each cluster using all other clusters as reference. Cluster defining markers are highlighted in bold.

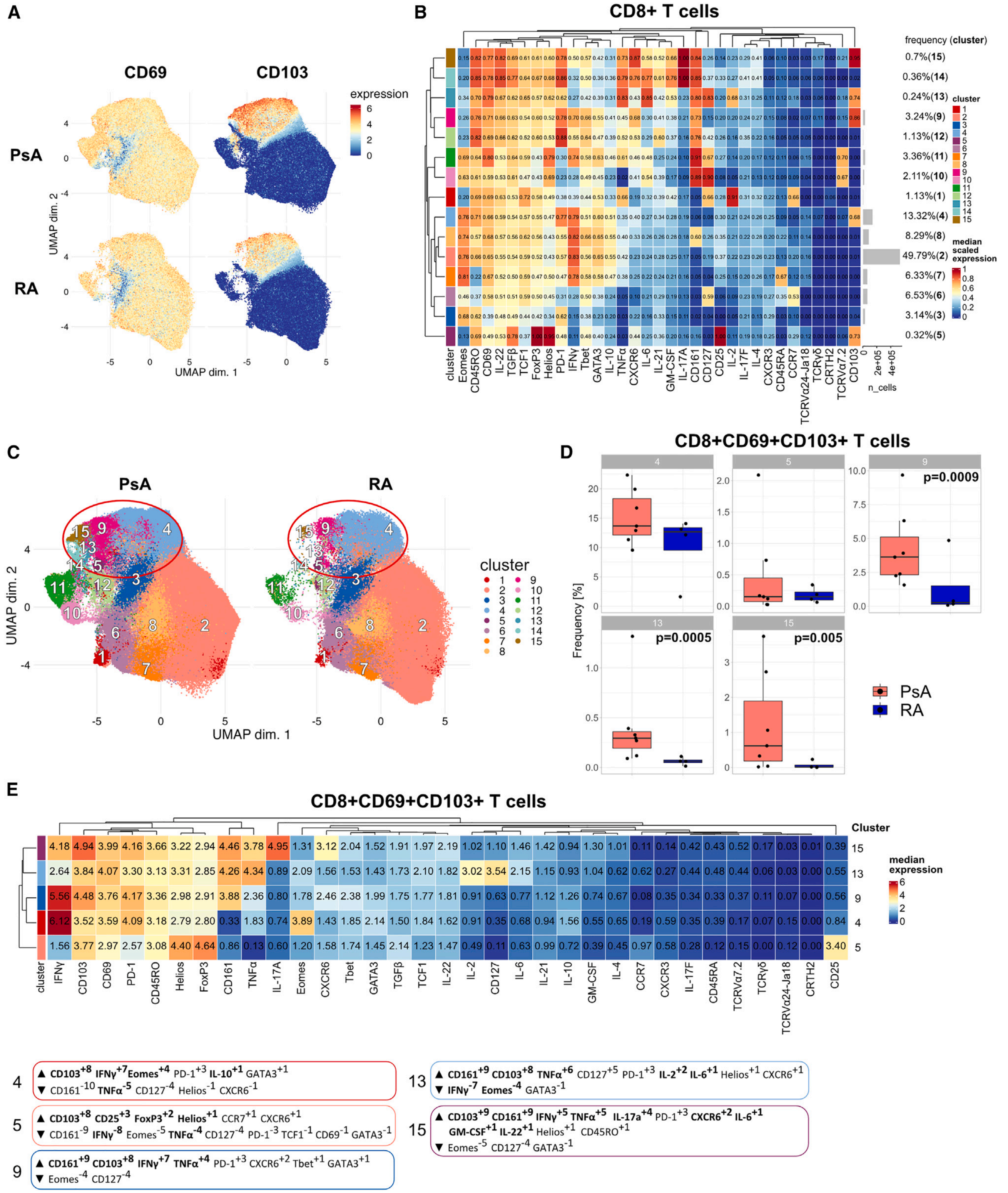

Figure 3. Cytokine expression profiles of synovial CD8+CD69+CD103+ T_{RM} cell populations

Synovial fluid CD3+ T cells were stimulated for 3 h with PMA/ionomycin in the presence of GolgiStop and stained with panel II; live CD8+ T cells were gated and analyzed.

(legend continued on next page)

with PsA, with distinct phenotypes and expression of effector and cytotoxic molecules. To analyze these cells in greater detail, we performed scRNA-seq of PsA synovial CD8+ T cells (n = 4; Table 1 for demographic and clinical information, and Table S1 for cell and VDJ recoveries). Cells were labeled with CITE-Seq antibodies to allow for integration of cellular protein and transcriptome measurements of T_{RM} cell markers. This approach confirmed our CyTOF data that while the majority of the synovial CD8+ T cells expressed CD69 at both the gene and protein levels, only a limited fraction of the cells expressed CD103 (Figure 4A).

Cell population identification by Seurat clustering resolved the CD8+ T cells into 18 distinct clusters (Figure 4B), similarly represented in all the donors (Figures S3A–S3C) and defined by differentially expressed genes (Figure S4 and Table S2). Clusters 4, 6, and 13 were identified as CD8+ T_{RM} populations based on matched gene and protein co-expression for CD69 and CD103 (Figure 4C). However, while clusters 4 and 6 showed a significant positive enrichment for a human T_{RM} cell signature[15] when compared with all other clusters, cluster 13 did not show the same significant enrichment (Figure 4D). Considering that cluster 13 also showed lower expression for CD103 (Figure 4C), we hypothesized that cluster 13 contained a proportion of T_{RM} cells, without these, however, being a distinct population. In addition, clusters 2, 3, 9, 12, 15, and 17, while negative for CD103, expressed CD69 in combination with significant upregulation of at least one other marker associated with tissue residency (CXCR6, CD49a, CD101, and PD-1), low expression of markers required for tissue egress (CD62L, S1PR1, and KLF2), and +/− low expression of CX3CR1 (Figure S3D), thus raising the possibility that they could represent CD103− T_{RM} cell populations. However, clusters 2, 15, and 17 were identified as mucosal-associated invariant T (MAIT) cells (based upon upregulated expression of TRAV1-2; Table S2), and clusters 3, 9, and 12 were not significantly enriched for the human T_{RM} cell signature[15] (Figure S3E).

To further characterize the CD8+CD69+CD103+ T_{RM} clusters, we interrogated the scRNA-seq dataset for the signatures identified by our CyTOF MEM analysis (Figure 2B). This revealed that cluster 4 showed a signature comparable to the cytotoxic effector T_{RM} cells, as indicated by expression of *PRF1*, *GZMA*, and *GZMB*, while cluster 6 possessed a type 17-like T_{RM} profile with expression of CD161 and CCR6 (Figure 4E). Cluster 13 showed a similar cytotoxic profile to cluster 4 but with high expression for *MKI67*, suggesting that these cells were highly proliferating. This was confirmed by a functional enrichment analysis showing highly significant enrichment for mitotic and cell cycle gene sets (Figure S5A). Since T_{RM} cells are typically shown to be non-cycling,[15] these data supported our hypothesis that, while containing a small proportion of T_{RM} cells, cluster 13 consisted mostly of cycling cells.

We performed differential gene expression analysis of each T_{RM} cluster compared to all other clusters (T_{RM} and CD8+CD103− T cells). This further defined a specific signature for cluster 4 characterized by high expression ($q < 0.05$) of multiple cytotoxicity-associated genes including *GZMA*, *GZMB*, *GZMH*, *GZMK*, *NKG7*, and *PRF1* together with T_{RM} canonical genes *ITGAE* (which encodes CD103), *PDCD1*, *LAG3*, and *CTLA4* (upregulated) and *SELL*, *KLF2*, and *KLF3* (downregulated). Cluster 6 instead showed a type 17-like signature with upregulation ($q < 0.05$) of canonical type 17 genes including *KLRB1*, *RORA*, *AHR*, *BATF*, and *CCR6*, together with the same T_{RM} canonical genes found differentially expressed in cluster 4 but with the addition of *S1PR1* and *EOMES* (downregulated) and *CXCR6* and *ZNF683* (upregulated) (Figure 4F; Table S3). Furthermore, although the expression of most cytokine genes was low due to lack of prior activation with either anti-CD3/CD28 monoclonal antibody (mAb) or PMA/ionomycin stimulation, we found that the few cells expressing typical type 17-related genes *IL17A*, *IL21*, and *IL26* were enriched in cluster 6 (Figure S5B). Genes encoding for *CXCL13*, *ALOX5AP*, and *LGALS1* were among the most differentially upregulated genes (fold change [FC] > 1.5 and $q < 0.05$) in both T_{RM} clusters when compared with all other clusters (Figure 4F). Functional enrichment analysis validated our classification by revealing significant enrichment for multiple cytotoxicity gene sets in cluster 4 and type 17 cell signature gene sets in cluster 6, together with ones associated with immune responses and T cell activation, which were shared by both T_{RM} clusters (Figure S5C).

To evaluate specific differences in the transcriptomic signatures between the two T_{RM} populations, we performed pairwise differential gene expression analyses between clusters 4 and 6. We identified distinct signatures, which matched with the T_{RM} profiles we obtained from our CyTOF data: cluster 4 showed a signature matching the cytotoxic effector profile characterized by significantly higher expression of perforin and granzymes as well the transcription factor Eomes, while cluster 6 matched the CD161+CCR6+ type 17-like T_{RM} cell population (Figures 4G, S5D; Table S3). Furthermore, when comparing the T_{RM} clusters, we found that cluster 4 was significantly enriched for a signature of SF CD8+HLA-DR^{hi} cells (Figure 4H), a population previously described in patients with PsA, which, despite not expressing *ITGAE* in that study, was also shown to have high expression of multiple HLA genes.[27] Conversely, cluster 6 showed a highly

(A) UMAPs based on the arcsinh-transformed expression of 33 markers evaluated in total CD8+ T cells from patients with PsA (n = 7) or RA (n = 4). From each sample, 38,000 cells were randomly selected. UMAPs show cell expression levels of CD69 (left column) and CD103 (right column), stratified by disease: PsA (top row) and RA (bottom row). Cells were clustered based on surface and intracellular markers.

(B) Heatmap of the median marker intensities of the 33 markers across the 15 cell populations obtained with FlowSOM as in Figure 1.

(C) UMAPs generated as in (A) stratified by disease and showing cells colored according to the 15 cell populations obtained with FlowSOM and characterized in (B); red circles highlight T_{RM} cell clusters.

(D) Cumulative data showing the relative abundance of CD8+CD69+CD103+T_{RM} cell clusters from (B), identified by co-expression of CD69 and CD103, in patients with PsA (red) and RA (blue). Boxplots show median ± IQR; data analyzed by GLMMs and $p < 0.05$ reported.

(E) Heatmap of the median marker intensities of the 33 markers across the 5 T_{RM} cell populations from (B). MEM labels (boxed) listed for each of the 5 CD8+CD69+CD103+ T_{RM} cell populations as described in Figure 2.

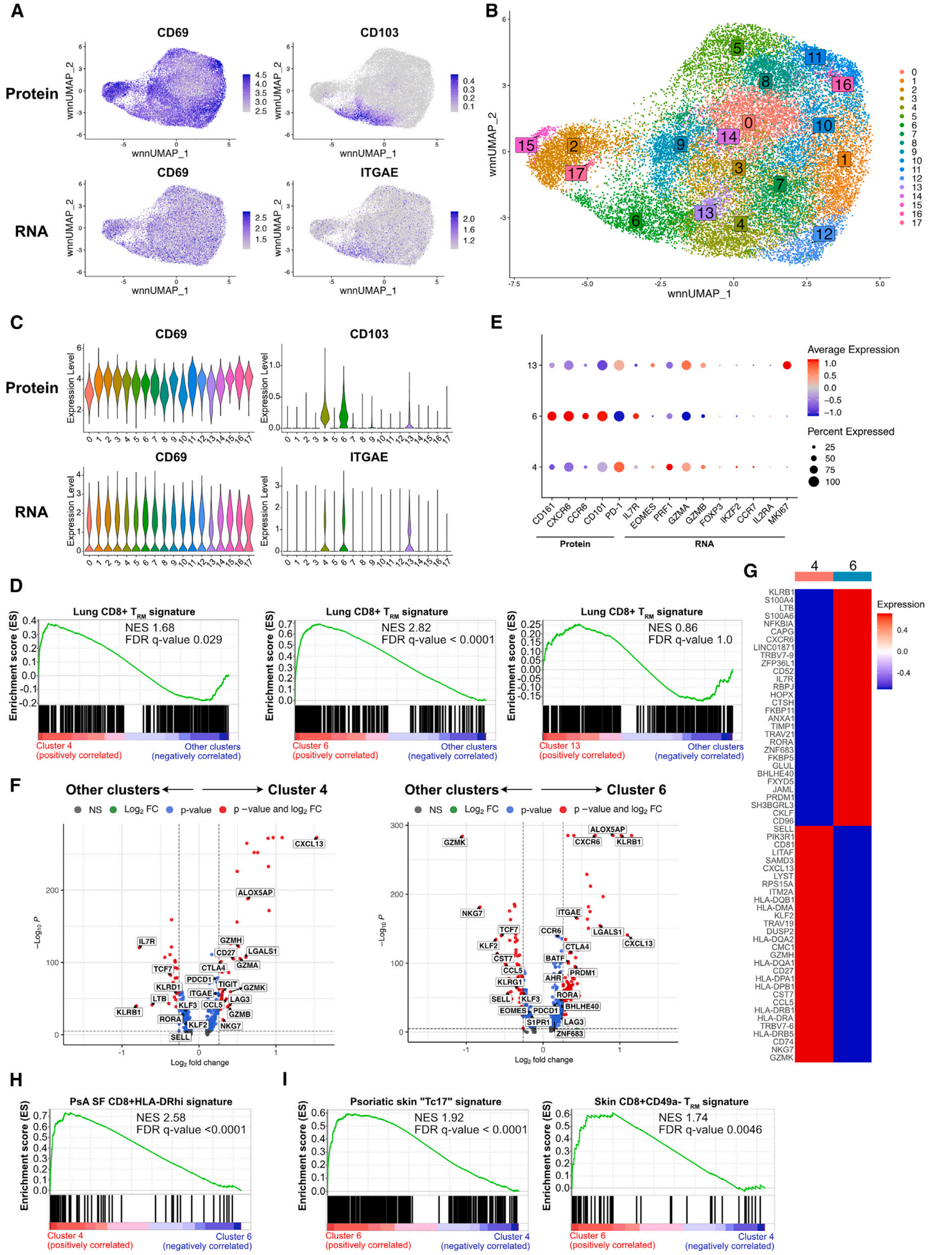

(legend on next page)

significant positive enrichment for a signature of *ITGAE*-expressing Tc17 cells found in psoriatic skin[28] and for skin CD8+CD49a− T_{RM} cells, which also have a type 17 phenotype and are enriched in psoriatic skin[14] (Figure 4I). This suggested that the population of type 17-like T_{RM} cells we identified in PsA SF showed similar characteristics to T_{RM} cells found in the skin, both in health and disease. Recent studies in mice have reported that T_{RM} cells from different tissues exhibit differential requirements for TGFβ signaling for differentiation and functionality.[23] Cluster 6, but not cluster 4, was significantly enriched for signatures of *ex vivo* TGFβ-stimulated human CD8+ T cells[29] (Figure S5E), and both TGFβ1 and TGFβR2 were modestly but significantly upregulated in cluster 6 compared with cluster 4 (Table S3), suggesting activation of the TGFβ signaling pathway in type 17-like but not cytotoxic synovial T_{RM} cells in PsA.

Synovial CD8+CD69+CD103+ T_{RM} cells from patients with PsA are polyclonal and show little clonal similarity to CD8+CD103− T cells

To assess the clonality of synovial CD8+CD69+CD103+ T_{RM} cells in patients with PsA and determine whether the TCR repertoire was shared between these T_{RM} and CD8+CD103− T cells, we used VDJ sequencing and mapped paired α/β-chain TCR sequences to gene expression for the same cells. We calculated the inverse Pielou clonality score for clusters that had at least 100 cells with paired gene expression and TCR sequences from each donor and found that all of these clusters were similarly polyclonal, as indicated by a low score (Figure 5A). We next calculated the Morisita similarity index to measure TCR composition overlap between the different clusters and found that in every donor, only a few clusters showed a degree of repertoire overlap, i.e., a Morisita similarity index score >0.4 (Figure S6A). While cluster 4 showed a modest degree of clonal overlap (median Morisita score > 0.4) with the CD8+CD103− T cell clusters 1, 7, and 12, cluster 6 displayed very limited repertoire sharing with either the other CD8+CD103− clusters or T_{RM} cluster 4 (Figures 5B and 5C). Clonotype analysis further confirmed that while cluster 4 shared a proportion of its top 10 clones with the CD8+CD103− T cell clusters 1, 7, and 12 (consistent with the higher Morisita score), cluster 6 showed little sharing, indicating a more distinct repertoire (Figure 5D). Increased clonotype similarity between T_{RM} cluster 4 and CD8+CD103− T cell clusters 1, 7, and 12 was associated with similar gene signatures characterized by high expression of cytotoxic markers including multiple HLAs and granzymes (Figure S6B). Taken together, these data demonstrate that SF-derived CD8+ T cells from patients with PsA generally display a polyclonal repertoire with a more distinct repertoire for type 17-like T_{RM} cells.

Synovial CD8+CD103− T cells of patients with PsA also show a prevalent type 17-like phenotype

Finally, we sought to explore whether we could find differences among the synovial CD8+CD103− T cell subsets in our patient cohorts using our CyTOF data. Among the clusters identified by FlowSOM and ConsensusClusterPlus from Figure 1C, we found that within CD8+CD103− T cells, clusters 9 and 10 were significantly enriched in patients with PsA, while cluster 2 was significantly enriched in patients with RA (Figure 6A). Within CD4+CD103− T cells, clusters 1 and 4 were significantly enriched in patients with PsA, while clusters 5, 6, and 9 were significantly enriched in patients with RA (Figure 6B).

MEM analysis of CD8+CD103− T cells in PsA-enriched clusters 9 and 10 revealed a similar type 17-like signature characterized by CD161, CD127, and CCR6 enrichment (Figure 6C); however, in cluster 9, there was specific enrichment for TCRVα7.2, identifying this cluster as MAIT cells, while cluster 10 resembled the IL-17A-expressing cluster 14 in Figure 3B and was therefore identified as conventional Tc17 cells. Conversely, cluster 2, enriched in patients with RA, showed Eomes, perforin, granzyme B, and CD127 enrichment, indicating a signature for cytotoxic effector cells. These data indicate that also in the CD8+CD103− T cells, there is a type 17-enriched CD8+ T cell signature in patients with PsA compared with a cytotoxic CD8+ T cell profile in RA.

MEM analysis of CD4+CD103− T cells in PsA-enriched clusters 1 and 4 revealed two distinct signatures. Cluster 1 showed positive enrichment for CD25, FoxP3, and Helios and negative enrichment for CD127, suggesting a Treg signature. Cluster 4 showed a Th17-like signature characterized by positive enrichment for CD161, CD127, and CCR6 (Figure 6D). Conversely, the RA-enriched clusters 5 and 6 showed similar signatures characterized by the enrichment for activation and proliferation markers CD25 and Ki67, granzymes A and B, CD161, CXCR6, and T-bet, with cluster 6 also enriched for CD49a and CD101. Finally, RA-enriched cluster 9 represented actively proliferating cells as demonstrated by the enrichment for Ki67 (Figure 6D).

In line with our findings when analyzing the phenotype of the T_{RM} cell populations, we found only minor phenotypic differences in the CD8+CD103− and CD4+CD103− populations

Figure 4. scRNA-seq analysis defined a distinct signature for T_{RM} populations in the synovial fluid of patients with PsA

SFMC samples from patients with PsA (n = 4) were sequenced independently using the 10× protocol.

(A) UMAPs showing cell protein (top row) and gene (bottom row) expression levels of CD69 (left) and CD103 (right) in CD8+ T cells from patients with PsA.

(B) UMAP showing cells colored according to the 18 populations obtained after Seurat clustering.

(C) Violin plots showing cell protein (top row) and gene (bottom row) expression levels of CD69 (left) and CD103 (right) in the 18 Seurat clusters.

(D) GSEA plots for lung CD8+ T_{RM} signature, comparing clusters 4 (left), 6 (middle), and 13 (right) with all other clusters; n = 4 per group. Normalized enrichment score (NES) and multiple test-adjusted q value from GSEA are shown.

(E) Dotplot showing either protein or gene expression of signature markers that defined distinct T_{RM} populations by CyTOF, across the identified 3 CD69+CD103+ clusters from (C). Dotplot heatmap showing the average expression (color) and percentage of cells (size) expressing the indicated genes or proteins.

(F) Volcano plots showing significant genes differentially expressed in cluster 4 (left) or 6 (right) compared with all other clusters. Differentially expressed genes were calculated using Wilcoxon rank-sum tests using the FindMarkers function in Seurat and shown in blue ($q < 0.05$) or red ($q < 0.05$ with a ± 1.2-fold change).

(G) Heatmap showing the normalized average expression of top 30 upregulated and downregulated genes ($q < 0.05$) in cluster 6 compared with cluster 4.

(H and I) GSEA plots comparing cluster 4 with cluster 6 for PsA synovial fluid (SF) CD8+HLA-DR^{hi} signature (H) and cluster 6 with cluster 4 for psoriatic skin Tc17 and skin CD8+CD49− T_{RM} signatures (I); n = 4 per group.

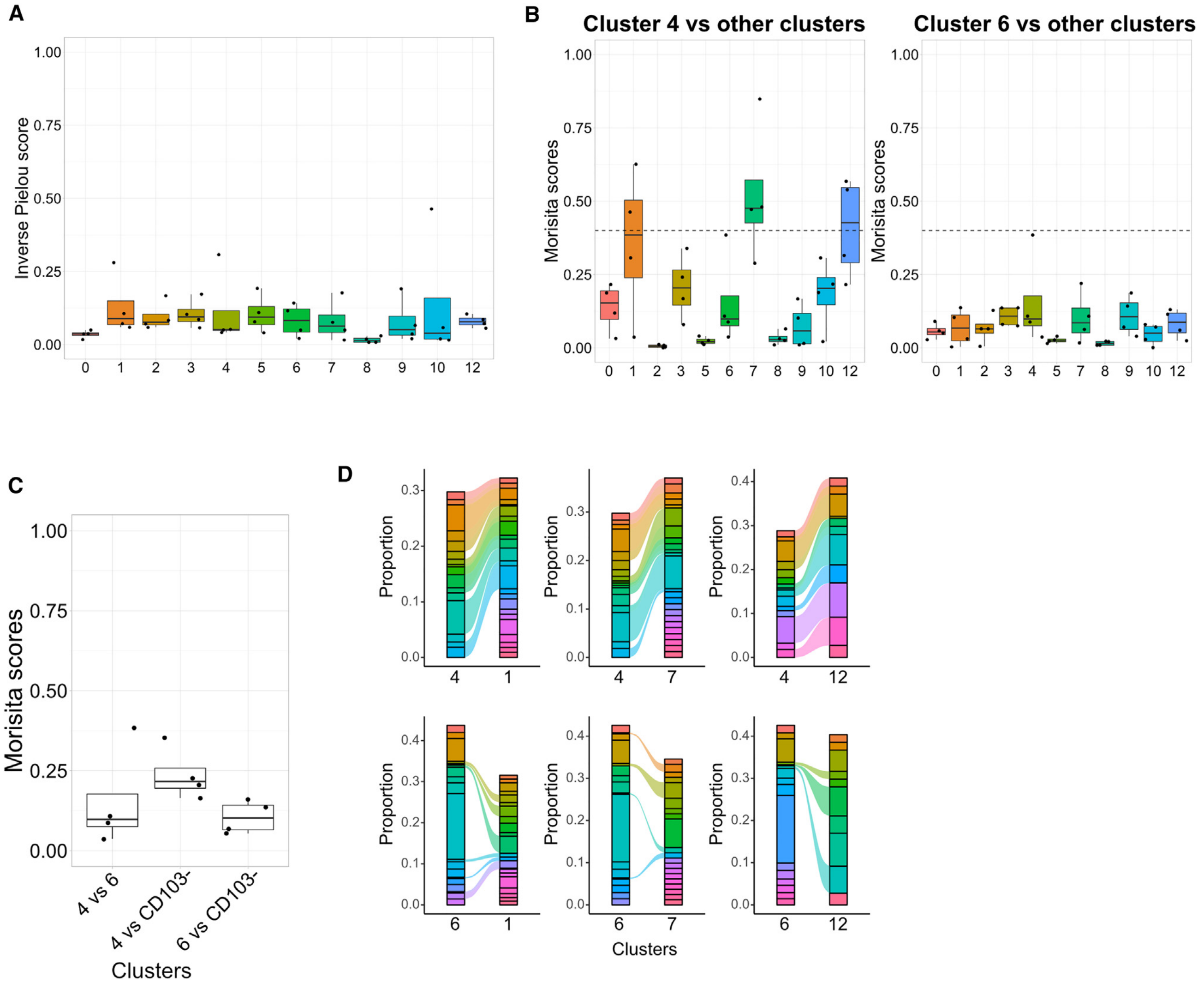

Figure 5. Limited clonal overlap in synovial CD8+ T cells from patients with PsA

(A) Cumulative data showing the inverse Pielou clonality score in every cluster from Figure 4B, which contains >100 TCR sequences for each donor (n = 4); Inverse Pielou score ranges from 0, which indicates a highly polyclonal population, to 1, which indicates a monoclonal population; boxplots show median ± IQR.

(B) Cumulative data showing the Morisita scores for cluster 4 (left) or 6 (right) compared with other clusters (n = 4); boxplots show median ± IQR; discretionary dotted line was drawn at score = 0.4 for comparison of scores.

(C) Cumulative data showing the Morisita scores for comparisons of T_{RM} clusters repertoires and of each T_{RM} cluster with CD8+CD103− T cell clusters (n = 4); boxplots show median ± IQR.

(D) Representative alluvial plots showing the proportion of the top 10 clones shared between clusters; alluvials connecting clusters are colored according to shared clones.

between patients with PsA or RA (Figures S7A and S7B). Taken together, these data demonstrate significant differences in the cellular composition of synovial T cells in patients with PsA versus RA, with evidence of a significant enrichment of both CD8+CD103+ T_{RM} and CD8+CD103− T cell subsets with a predominant type 17-like signature in patients with PsA.

DISCUSSION

The high-dimensional immune profiling study presented here deeply phenotyped in parallel synovial CD8+ and CD4+ CD69+ CD103+ T_{RM} cells in two rheumatic diseases and identified them as a heterogeneous population characterized by multiple distinct phenotypes and functions.

It was previously reported that CD8+ T_{RM} cells, despite sharing a core transcriptional signature, exhibit both inter- and intra-organ heterogeneity and can differ in terms of CD103 expression, cytokine production, cytotoxic potential, proliferative capacity, metabolism, and commitment to tissue residency.[8,14,23,30–32] Our CyTOF analysis revealed a type 17-like CD8+CD69+CD103+ T_{RM} cell population (cluster 12) that was found at a higher frequency in the joints of patients with PsA

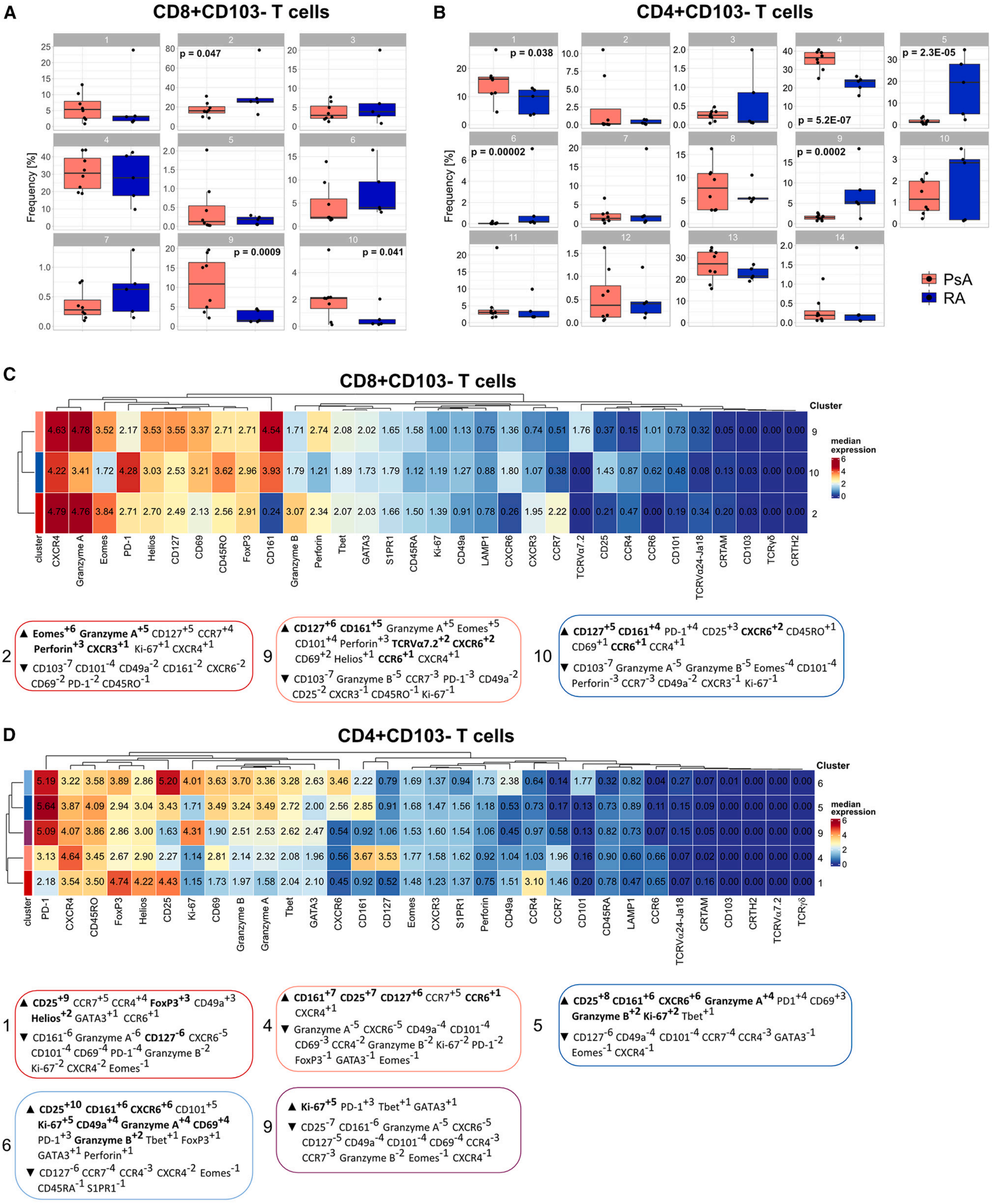

(legend on next page)

and was virtually absent in patients with RA. This cluster showed a classical type 17 phenotype, characterized by the expression of CD161 and CCR6, together with high expression of CXCR6, but no markers of cytotoxicity. When we phenotyped the cells after PMA/ionomycin stimulation to assess cytokine production, we were able to identify an equivalent type 17-like T_{RM} cell population (cluster 15), characterized by expression of both IL-17A and IFNγ. Additionally, we found two more clusters (9 and 13) with similar type 17-like profiles, including high CD161 expression, which identifies IL-17-producing cells,[33] but with different combinations of cytokines; this difference might be attributed to the stochastic nature of cytokine expression or represent a different functional stage of type 17-like T_{RM} cells captured in response to PMA/ionomycin stimulation. Nonetheless, all three clusters were found significantly enriched in patients with PsA, suggesting that these clusters represent different fates or states of type 17-like T_{RM} cells. These findings are consistent with previous work by us and others showing that Tc17/T_{RM} cells in PsA are polyfunctional and able to produce multiple cytokines, which drive PsA inflammation.[21,34] scRNA-seq validated the presence of a distinct population of type 17-like CD8+CD69+CD103+ T_{RM} cells in PsA SF (cluster 6) and defined a unique gene signature for these cells that set them apart from other T_{RM} cells with significant upregulation of canonical type 17 signature genes including *KLRB1*, *RORA*, *AHR*, *BATF*, and *CCR6* as well as expression of *IL17A*, *IL21*, and *IL26*. The type 17-like signature, including the ability to produce IL-17A and other inflammatory cytokines, low cytotoxicity, and a distinct TCR repertoire compared with other T_{RM} and CD8+CD103− T cells all point to this being a unique population of T_{RM} cells with a discrete function.

Our scRNA-seq analysis also suggested striking similarities between type 17-like synovial T_{RM} cells in PsA and T_{RM} cells from the skin including (1) common expression of specific metabolic genes including *BHLHE40* that is upregulated in IL-17-producing T_{RM} cells in human psoriatic skin[28]; (2) an enrichment for signatures of *ITGAE*+ type 17 T_{RM} cells from psoriatic lesions[28] and of CD49a− T_{RM} cells that are enriched in psoriatic lesions[14]; and (3) a specific enrichment in the type 17 (cluster 6), but not cytotoxic (cluster 4), T_{RM} cells for a signature of TGFβ stimulation, which has been shown to be required for the differentiation and maintenance of T_{RM} cells in mice skin. Thus, the specific enrichment of type 17-like T_{RM} cells in the SF of patients with PsA and their similarity to T_{RM} cells from the skin in psoriasis point to a specific role for type 17 T_{RM} cells in PsA. In this context, it is interesting to note that in psoriasis, IL-17-producing resident T cells were shown to persist in "normal" skin at the sites of healed psoriasis at frequencies that inversely correlated with time to relapse in patients who stopped treatment.[35,36] Thus, it was hypothesized that skin-resident IL-17-producing T_{RM} cells may drive relapses in psoriasis. One could speculate that the synovial type 17 T_{RM} cells we describe in the current study may have a similar role in driving joint flares in PsA.

Our study also identified cytotoxic CD8+ T_{RM} cells in the inflamed joint present at similar levels in both PsA and RA. CyTOF analysis identified three populations of CD8+ cytotoxic T_{RM} cells (clusters 8, 14, and 15) characterized by high expression of granzyme A, granzyme B, and perforin, together with Eomes and PD1, indicating a cytotoxic effector profile. However, despite this segmentation into multiple clusters due to differences in expression of markers including CD101 and CD49a, stimulation with PMA/ionomycin revealed that these cells uniformly responded by secreting high levels of IFNγ; this suggested a specialized function of these cells despite phenotypic heterogeneity. Indeed, our scRNA-seq analysis identified a single signature for cytotoxic T_{RM} cells (cluster 4) that resembled a previously described PsA SF-derived CD8+HLA-DR^{hi} T cell population.[27] While in that study, *ITGAE* was not found to be differentially expressed in any of the CD8+ T cell clusters (the authors did not include CITE-Seq antibodies for CD103), our gene set enrichment analysis (GSEA) showed a significant positive enrichment for this HLA class II-associated gene signature in our cytotoxic CD103+ and *ITGAE*+ T_{RM} cell population. Furthermore, a similar HLA class II signature has been identified in highly cytotoxic, IFNγ-producing CD8+ T cells from the peripheral blood of healthy volunteers,[37] which also expressed high levels of *CCL5*, similarly to cluster 4 T_{RM} cells. Together with their polyclonal nature as well as their TCR sharing with other cytotoxic non-T_{RM} cell populations, these data suggest a specialized cytotoxic effector function for cluster 4 T_{RM} cells.

The commonly used core signature of T_{RM} cells (e.g., CD103, CD69, CXCR6, PD-1, CD49a, and CD101) may not accurately reflect the heterogeneity within T_{RM} cells at different tissue sites or identify potential T_{RM} subset-specific associations to pathological conditions. Our data show that cytotoxic and type 17-like T_{RM} cells differ in terms of cytokine production, cytotoxic potential, and TGFβ "priming." The gene signatures for cytotoxic and type 17-like T_{RM} cells defined in this study may allow for improved T_{RM} cell population segmentation within tissues and thus help identify changes in T_{RM} cell composition at different sites and in different diseases for future studies.

Our CyTOF analysis also identified a Treg-like CD8+ T_{RM} population (cluster 13) that was present at low frequencies in both PsA and RA. This T_{RM} cell population expressed all the markers that are associated with CD4+ Treg cells including high levels of CD25, Foxp3, and Helios as well as low CD127; we, however, also detected expression of CD161 and CCR6, albeit at low levels, which suggests a type 17 profile. We and others have previously characterized a distinct population of human CD4+ Treg cells with similarly unique type 17-like characteristics and functions, which we found enriched in the SF of patients with RA as well as in the lamina propria of patients with Crohn's disease.[38–41] Furthermore, a recent scRNA-seq study has described a population of synovial CD8+CD161+ Treg cells with a distinct molecular signature in patients with PsA.[42] Our

Figure 6. Enrichment of conventional type 17 CD8+ T cells in the SF of patients with PsA

(A and B) Cumulative data showing the relative abundance of CD8+ (A) and CD4+ (B) CD103− T cell clusters from Figure 1C in patients with PsA (n = 8, red) or RA (n = 5, blue). Boxplots show median ± IQR; data analyzed by GLMMs and $p < 0.05$ reported.

(C and D) Heatmap and MEM modeling of the median marker intensities of the 32 markers across the significantly enriched (in either PsA or RA) CD8+CD103− (C) or CD4+CD103− (D) cell populations from (A and B). Heatmap and MEM details as described in Figures 1 and 2.

data show that this CD8+ Treg-like population harbors various markers of T_{RM} cells, suggesting that these cells may persist in the synovial joints of patients with PsA and RA. It remains to be established whether these cells possess the same functional characteristics as their CD4+ counterparts, an issue that is challenging to address given their low frequency in the SF and therefore their absence in our scRNA-seq data. However, based on their type 17-like signature and similarity to their CD4+ counterparts, one could hypothesize that these cells are specialized to specifically suppress local type 17 responses, as was previously shown for CD4+ Treg cells in mice.[43]

Contrary to our initial hypothesis, we did not detect major qualitative differences in the phenotypes of the CD8+ and CD4+ T_{RM} cell populations between PsA and RA. This suggests that synovial T cells in these two diseases may differ more in terms of the composition and frequency of specific subsets rather than in their phenotypes. In addition, and concordant with previous work,[21,22,44,45] we found that within CD103− T cells in PsA SF, CD8+ MAIT cells and conventional Tc17-like (CD8+CD161+CCR6+) cells were significantly enriched, as well as CD4+ Treg and Th17-like (CD4+CD161+CCR6+) cells, while cytotoxic CD8+ and cytotoxic/proliferative CD4+ T cells were significantly enhanced in RA. Taken together, these data reveal significant quantitative differences in certain CD8+ and CD4+ T cell populations between PsA and RA.

To conclude, we have characterized three distinct CD8+ CD69+CD103+ T_{RM} cell populations within the inflamed arthritic joint: cytotoxic and Treg-like T_{RM} cells, which are present in the synovial joint of patients with both PsA and RA, while type 17-like T_{RM} cells, as well as type 17-like CD8+CD103− T cells, are specifically enriched in the synovial joint of patients with PsA. These data extend our previous findings[21] and shed light on a potential underlying cause for the difference in clinical efficacy of IL-17A blockade in PsA versus RA[46]: a significantly larger fraction of IL-17A-secreting tissue-resident and non-resident CD8+ T cells within the synovial PsA joint may contribute to a larger, and perhaps a more unique, extent to the immunopathology and persistence of this disease.

Limitations of the study

One limitation of our study is the patient heterogeneity. While no significant bias or association due to patient clinical parameters, sex/gender, age, disease duration, or treatment strategy was observed in the data analysis, we cannot draw definitive conclusions in this regard due to the relatively small sample sizes.

In this study, we focused on T_{RM} cells that were defined by expression of CD69 and CD103 and enriched for a signature of T_{RM} cells from homeostatic human tissue.[15] It is known that in certain organs, for example the liver, the majority of T_{RM} cells do not express CD103,[47,48] and therefore these T_{RM} cells are defined by CD69 expression. However, while CD69 is considered an effective marker of tissue residency in the homeostatic state and is constitutively expressed by T_{RM} cells from all tissues, it is also an activation marker. As such, on its own, CD69 is not a reliable marker of tissue residency in inflamed tissue. This is supported by our finding that most synovial T cells from inflamed joints expressed CD69 at the protein and mRNA levels. We identified several clusters of CD69+CD103− T_{RM} cells in our datasets that expressed other markers associated with a T_{RM} cell phenotype but were not enriched for a T_{RM} signature. In the absence of consensus markers for CD103− T_{RM} cells in inflamed human tissue, we did not classify these cells as tissue-resident T cells and referred to these cells as CD103− T cells instead.

STAR★METHODS

Detailed methods are provided in the online version of this paper and include the following:

- KEY RESOURCES TABLE
- RESOURCE AVAILABILITY
  - Lead contact
  - Materials availability
  - Data and code availability
- EXPERIMENTAL MODEL AND SUBJECT DETAILS
  - Participant recruitment and ethical approval
- METHOD DETAILS
  - Cell isolation
  - CyTOF staining and sample acquisition
  - Cell sorting for single cell RNA sequencing
  - Single cell library preparation and sequencing
- QUANTIFICATION AND STATISTICAL ANALYSIS
  - CyTOF data analysis
  - Pre-processing of scRNAseq data
  - scRNAseq analysis
  - Single cell TCR sequencing analysis

SUPPLEMENTAL INFORMATION

Supplemental information can be found online at https://doi.org/10.1016/j.celrep.2023.112514.

ACKNOWLEDGMENTS

This study was supported by a Sanofi iAward (to G.A.M.P., B.W.K., and L.S.T.), a Versus Arthritis programme grant (ref. 21139 to L.S.T.), a KCL PhD studentship (to E.H.G.), an MRC clinical research training fellowship (ref MR/P018904/1 to L.E.D.), and the Department of Health via the National Institute for Health Research (NIHR) comprehensive Biomedical Research Centre (BRC) award to Guy's & St. Thomas' NHS Foundation Trust in partnership with KCL and King's College Hospital NHS Foundation Trust. The views expressed are those of the author(s) and not necessarily those of the NHS, the NIHR, or the Department of Health.

AUTHOR CONTRIBUTIONS

L.S.T., K.J.A.S., B.W.K., G.A.M.P., and L.E.D. contributed to study conceptualization and design; G.A.M.P. and E.H.G. developed the CyTOF antibody panels and performed CyTOF and scRNA-seq wet-lab experiments; S.L. aided CyTOF staining and data acquisition; G.A.M.P. performed CyTOF data analysis; L.E.D. performed scRNA-seq analysis with bioinformatic support from S.K., P.D., M.J.P., and G.A.M.P. G.A.M.P., L.E.D., E.H.G., T.L., and L.S.T. interpreted the data; B.W.K. was responsible for patient recruitment; samples were processed and cataloged by S.E.R., S.L., and E.H.G.; G.A.M.P., L.E.D., E.H.G., and L.S.T. wrote the manuscript; and L.S.T. supervised the overall project. All of the authors reviewed and approved the final version of the manuscript.

DECLARATION OF INTERESTS

This study was supported in part by a Sanofi iAward to G.A.M.P., B.W.K., and L.S.T. T.L. is an employee of Sanofi. L.S.T. has previously received speaker fees and/or research support from GSK, Novo Nordisk A/S, UCB, and Novartis, outside this work. B.W.K. has received research support from Eli-Lilly, Novartis, Roche Pharmaceuticals, and UCB Pharma and has been an advisor or received speaker fees from Eli-Lilly, Janssen, and Novartis, outside this work.

Received: October 25, 2022
Revised: February 21, 2023
Accepted: May 1, 2023
Published: May 16, 2023

REFERENCES

1. Veale, D.J., and Fearon, U. (2015). What makes psoriatic and rheumatoid arthritis so different? RMD Open *1*, e000025. https://doi.org/10.1136/rmdopen-2014-000025.
2. Masopust, D., and Soerens, A.G. (2019). Tissue-resident T cells and other resident leukocytes. Annu. Rev. Immunol. *37*, 521–546. https://doi.org/10.1146/annurev-immunol-042617-053214.
3. Szabo, P.A., Miron, M., and Farber, D.L. (2019). Location, location, location: tissue resident memory T cells in mice and humans. Sci Immunol *4*. https://doi.org/10.1126/sciimmunol.aas9673.
4. Mueller, S.N., and Mackay, L.K. (2016). Tissue-resident memory T cells: local specialists in immune defence. Nat. Rev. Immunol. *16*, 79–89. https://doi.org/10.1038/nri.2015.3.
5. Sathaliyawala, T., Kubota, M., Yudanin, N., Turner, D., Camp, P., Thome, J.J.C., Bickham, K.L., Lerner, H., Goldstein, M., Sykes, M., et al. (2013). Distribution and compartmentalization of human circulating and tissue-resident memory T cell subsets. Immunity *38*, 187–197. https://doi.org/10.1016/j.immuni.2012.09.020.
6. Iijima, N., and Iwasaki, A. (2015). Tissue instruction for migration and retention of TRM cells. Trends Immunol. *36*, 556–564. https://doi.org/10.1016/j.it.2015.07.002.
7. Klicznik, M.M., Morawski, P.A., Hollbacher, B., Varkhande, S.R., Motley, S.J., Kuri-Cervantes, L., Goodwin, E., Rosenblum, M.D., Long, S.A., Brachtl, G., et al. (2019). Human CD4(+)CD103(+) cutaneous resident memory T cells are found in the circulation of healthy individuals. Sci Immunol *4*. https://doi.org/10.1126/sciimmunol.aav8995.
8. Fonseca, R., Beura, L.K., Quarnstrom, C.F., Ghoneim, H.E., Fan, Y., Zebley, C.C., Scott, M.C., Fares-Frederickson, N.J., Wijeyesinghe, S., Thompson, E.A., et al. (2020). Developmental plasticity allows outside-in immune responses by resident memory T cells. Nat. Immunol. *21*, 412–421. https://doi.org/10.1038/s41590-020-0607-7.
9. Stolley, J.M., Johnston, T.S., Soerens, A.G., Beura, L.K., Rosato, P.C., Joag, V., Wijeyesinghe, S.P., Langlois, R.A., Osum, K.C., Mitchell, J.S., and Masopust, D. (2020). Retrograde migration supplies resident memory T cells to lung-draining LN after influenza infection. J. Exp. Med. *217*. https://doi.org/10.1084/jem.20192197.
10. Strobl, J., Gail, L.M., Kleissl, L., Pandey, R.V., Smejkal, V., Huber, J., Puxkandl, V., Unterluggauer, L., Dingelmaier-Hovorka, R., Atzmuller, D., et al. (2021). Human resident memory T cells exit the skin and mediate systemic Th2-driven inflammation. J. Exp. Med. *218*. https://doi.org/10.1084/jem.20210417.
11. Zundler, S., Becker, E., Spocinska, M., Slawik, M., Parga-Vidal, L., Stark, R., Wiendl, M., Atreya, R., Rath, T., Leppkes, M., et al. (2019). Hobit- and Blimp-1-driven CD4+ tissue-resident memory T cells control chronic intestinal inflammation. Nat. Immunol. *20*, 288–300. https://doi.org/10.1038/s41590-018-0298-5.
12. Liu, Y., Wang, H., Taylor, M., Cook, C., Martinez-Berdeja, A., North, J.P., Harirchian, P., Hailer, A.A., Zhao, Z., Ghadially, R., et al. (2022). Classification of human chronic inflammatory skin disease based on single-cell immune profiling. Sci Immunol *7*, eabl9165. https://doi.org/10.1126/sciimmunol.abl9165.
13. Wiggins, B.G., Pallett, L.J., Li, X., Davies, S.P., Amin, O.E., Gill, U.S., Kucykowicz, S., Patel, A.M., Aliazis, K., Liu, Y.S., et al. (2022). The human liver microenvironment shapes the homing and function of CD4+ T-cell populations. Gut *71*, 1399–1411. https://doi.org/10.1136/gutjnl-2020-323771.
14. Cheuk, S., Schlums, H., Gallais Sérézal, I., Martini, E., Chiang, S.C., Marquardt, N., Gibbs, A., Detlofsson, E., Introini, A., Forkel, M., et al. (2017). CD49a expression defines tissue-resident CD8+ T cells poised for cytotoxic function in human skin. Immunity *46*, 287–300. https://doi.org/10.1016/j.immuni.2017.01.009.
15. Kumar, B.V., Ma, W., Miron, M., Granot, T., Guyer, R.S., Carpenter, D.J., Senda, T., Sun, X., Ho, S.-H., Lerner, H., et al. (2017). Human tissue-resident memory T cells are defined by core transcriptional and functional signatures in lymphoid and mucosal sites. Cell Rep. *20*, 2921–2934. https://doi.org/10.1016/j.celrep.2017.08.078.
16. Pallett, L.J., Davies, J., Colbeck, E.J., Robertson, F., Hansi, N., Easom, N.J.W., Burton, A.R., Stegmann, K.A., Schurich, A., Swadling, L., et al. (2017). IL-2high tissue-resident T cells in the human liver: sentinels for hepatotropic infection. J. Exp. Med. *214*, 1567–1580. https://doi.org/10.1084/jem.20162115.
17. Pizzolla, A., Nguyen, T.H., Sant, S., Jaffar, J., Loudovaris, T., Mannering, S.I., Thomas, P.G., Westall, G.P., Kedzierska, K., and Wakim, L.M. (2018). Influenza-specific lung-resident memory T cells are proliferative and polyfunctional and maintain diverse TCR profiles. J. Clin. Invest. *128*, 721–733. https://doi.org/10.1172/JCI96957.
18. Chang, M.H., Levescot, A., Nelson-Maney, N., Blaustein, R.B., Winden, K.D., Morris, A., Wactor, A., Balu, S., Grieshaber-Bouyer, R., Wei, K., et al. (2021). Arthritis flares mediated by tissue-resident memory T cells in the joint. Cell Rep. *37*, 109902. https://doi.org/10.1016/j.celrep.2021.109902.
19. Jung, J., Lee, J.S., Kim, Y.G., Lee, C.K., Yoo, B., Shin, E.-C., and Hong, S. (2020). Synovial fluid CD69+CD8+ T cells with tissue-resident phenotype mediate perforin-dependent citrullination in rheumatoid arthritis. Clin Transl Immunology *9*, e1140. https://doi.org/10.1002/cti2.1140.
20. Petrelli, A., Mijnheer, G., Hoytema van Konijnenburg, D.P., van der Wal, M.M., Giovannone, B., Mocholi, E., Vazirpanah, N., Broen, J.C., Hijnen, D., Oldenburg, B., et al. (2018). PD-1+CD8+ T cells are clonally expanding effectors in human chronic inflammation. J. Clin. Invest. *128*, 4669–4681. https://doi.org/10.1172/JCI96107.
21. Steel, K.J.A., Srenathan, U., Ridley, M., Durham, L.E., Wu, S.-Y., Ryan, S.E., Hughes, C.D., Chan, E., Kirkham, B.W., and Taams, L.S. (2020). Polyfunctional, proinflammatory, tissue-resident memory phenotype and function of synovial interleukin-17A+CD8+ T cells in psoriatic arthritis. Arthritis Rheumatol. *72*, 435–447. https://doi.org/10.1002/art.41156.
22. Argyriou, A., Wadsworth, M.H., 2nd, Lendvai, A., Christensen, S.M., Hensvold, A.H., Gerstner, C., van Vollenhoven, A., Kravarik, K., Winkler, A., Malmstrom, V., and Chemin, K. (2022). Single cell sequencing identifies clonally expanded synovial CD4(+) T(PH) cells expressing GPR56 in rheumatoid arthritis. Nat. Commun. *13*, 4046. https://doi.org/10.1038/s41467-022-31519-6.
23. Christo, S.N., Evrard, M., Park, S.L., Gandolfo, L.C., Burn, T.N., Fonseca, R., Newman, D.M., Alexandre, Y.O., Collins, N., Zamudio, N.M., et al. (2021). Discrete tissue microenvironments instruct diversity in resident memory T cell function and plasticity. Nat. Immunol. *22*, 1140–1151. https://doi.org/10.1038/s41590-021-01004-1.
24. Nowicka, M., Krieg, C., Crowell, H.L., Weber, L.M., Hartmann, F.J., Guglietta, S., Becher, B., Levesque, M.P., and Robinson, M.D. (2017). CyTOF workflow: differential discovery in high-throughput high-dimensional cytometry datasets. F1000Res *6*, 748. https://doi.org/10.12688/f1000research.11622.3.
25. Diggins, K.E., Greenplate, A.R., Leelatian, N., Wogsland, C.E., and Irish, J.M. (2017). Characterizing cell subsets using marker enrichment

CellPress
OPEN ACCESS

modeling. Nat. Methods *14*, 275–278. https://doi.org/10.1038/nmeth.4149.

26. Bromley, S.K., Akbaba, H., Mani, V., Mora-Buch, R., Chasse, A.Y., Sama, A., and Luster, A.D. (2020). CD49a regulates cutaneous resident memory CD8+ T cell persistence and response. Cell Rep. *32*, 108085. https://doi.org/10.1016/j.celrep.2020.108085.
27. Penkava, F., Velasco-Herrera, M.D.C., Young, M.D., Yager, N., Nwosu, L.N., Pratt, A.G., Lara, A.L., Guzzo, C., Maroof, A., Mamanova, L., et al. (2020). Single-cell sequencing reveals clonal expansions of pro-inflammatory synovial CD8 T cells expressing tissue-homing receptors in psoriatic arthritis. Nat. Commun. *11*, 4767-4711. https://doi.org/10.1038/s41467-020-18513-6.
28. Liu, J., Chang, H.-W., Huang, Z.-M., Nakamura, M., Sekhon, S., Ahn, R., Munoz-Sandoval, P., Bhattarai, S., Beck, K.M., Sanchez, I.M., et al. (2021). Single-cell RNA sequencing of psoriatic skin identifies pathogenic Tc17 cell subsets and reveals distinctions between CD8+ T cells in autoimmunity and cancer. J. Allergy Clin. Immunol. *147*, 2370–2380. https://doi.org/10.1016/j.jaci.2020.11.028.
29. Dahmani, A., Janelle, V., Carli, C., Richaud, M., Lamarche, C., Khalili, M., Goupil, M., Bezverbnaya, K., Bramson, J.L., and Delisle, J.-S. (2019). TGFβ programs central memory differentiation in ex vivo-stimulated human T cells. Cancer Immunol Res *7*, 1426–1439. https://doi.org/10.1158/2326-6066.CIR-18-0691.
30. Kurd, N.S., He, Z., Louis, T.L., Milner, J.J., Omilusik, K.D., Jin, W., Tsai, M.S., Widjaja, C.E., Kanbar, J.N., Olvera, J.G., et al. (2020). Early precursors and molecular determinants of tissue-resident memory CD8+ T lymphocytes revealed by single-cell RNA sequencing. Sci Immunol *5*. https://doi.org/10.1126/sciimmunol.aaz6894.
31. Crowl, J.T., Heeg, M., Ferry, A., Milner, J.J., Omilusik, K.D., Toma, C., He, Z., Chang, J.T., and Goldrath, A.W. (2022). Tissue-resident memory CD8+ T cells possess unique transcriptional, epigenetic and functional adaptations to different tissue environments. Nat. Immunol. *1-11*. https://doi.org/10.1038/s41590-022-01229-8.
32. Milner, J.J., Toma, C., He, Z., Kurd, N.S., Nguyen, Q.P., McDonald, B., Quezada, L., Widjaja, C.E., Witherden, D.A., Crowl, J.T., et al. (2020). Heterogenous populations of tissue-resident CD8+ T cells are generated in response to infection and malignancy. Immunity *52*, 808–824.e807. https://doi.org/10.1016/j.immuni.2020.04.007.
33. Maggi, L., Santarlasci, V., Capone, M., Peired, A., Frosali, F., Crome, S.Q., Querci, V., Fambrini, M., Liotta, F., Levings, M.K., et al. (2010). CD161 is a marker of all human IL-17-producing T-cell subsets and is induced by RORC. Eur. J. Immunol. *40*, 2174–2181. https://doi.org/10.1002/eji.200940257.
34. Wade, S.M., Canavan, M., McGarry, T., Low, C., Wade, S.C., Mullan, R.H., Veale, D.J., and Fearon, U. (2019). Association of synovial tissue polyfunctional T-cells with DAPSA in psoriatic arthritis. Ann. Rheum. Dis. *78*, 350–354. https://doi.org/10.1136/annrheumdis-2018-214138.
35. Cheuk, S., Wikén, M., Blomqvist, L., Nylén, S., Talme, T., Ståhle, M., and Eidsmo, L. (2014). Epidermal Th22 and Tc17 cells form a localized disease memory in clinically healed psoriasis. J. Immunol. *192*, 3111–3120. https://doi.org/10.4049/jimmunol.1302313.
36. Gallais Sérézal, I., Classon, C., Cheuk, S., Barrientos-Somarribas, M., Wadman, E., Martini, E., Chang, D., Xu Landén, N., Ehrström, M., Nylén, S., and Eidsmo, L. (2018). Resident T cells in resolved psoriasis steer tissue responses that stratify clinical outcome. J. Invest. Dermatol. *138*, 1754–1763. https://doi.org/10.1016/j.jid.2018.02.030.
37. Nicolet, B.P., Guislain, A., van Alphen, F.P.J., Gomez-Eerland, R., Schumacher, T.N.M., van den Biggelaar, M., and Wolkers, M.C. (2020). CD29 identifies IFN-γ-producing human CD8+ T cells with an increased cytotoxic potential. Proc. Natl. Acad. Sci. USA *117*, 6686–6696. https://doi.org/10.1073/pnas.1913940117.
38. Povoleri, G.A.M., Nova-Lamperti, E., Scotta, C., Fanelli, G., Chen, Y.C., Becker, P.D., Boardman, D., Costantini, B., Romano, M., Pavlidis, P., et al. (2018). Human retinoic acid-regulated CD161(+) regulatory T cells support wound repair in intestinal mucosa. Nat. Immunol. *19*, 1403–1414. https://doi.org/10.1038/s41590-018-0230-z.
39. Afzali, B., Mitchell, P.J., Edozie, F.C., Povoleri, G.A.M., Dowson, S.E., Demandt, L., Walter, G., Canavan, J.B., Scotta, C., Menon, B., et al. (2013). CD161 expression characterizes a subpopulation of human regulatory T cells that produces IL-17 in a STAT3-dependent manner. Eur. J. Immunol. *43*, 2043–2054. https://doi.org/10.1002/eji.201243296.
40. Pesenacker, A.M., Bending, D., Ursu, S., Wu, Q., Nistala, K., and Wedderburn, L.R. (2013). CD161 defines the subset of FoxP3+ T cells capable of producing proinflammatory cytokines. Blood *121*, 2647–2658. https://doi.org/10.1182/blood-2012-08-443473.
41. Duurland, C.L., Brown, C.C., O'Shaughnessy, R.F.L., and Wedderburn, L.R. (2017). CD161+ tconv and CD161+ Treg share a transcriptional and functional phenotype despite limited overlap in TCRβ repertoire. Front. Immunol. *8*, 103. https://doi.org/10.3389/fimmu.2017.00103.
42. Simone, D., Penkava, F., Ridley, A., Sansom, S., Al-Mossawi, M.H., and Bowness, P. (2021). Single cell analysis of spondyloarthritis regulatory T cells identifies distinct synovial gene expression patterns and clonal fates. Commun Biol *4*, 1395-1315. https://doi.org/10.1038/s42003-021-02931-3.
43. Chaudhry, A., Rudra, D., Treuting, P., Samstein, R.M., Liang, Y., Kas, A., and Rudensky, A.Y. (2009). CD4+ regulatory T cells control TH17 responses in a Stat3-dependent manner. Science *326*, 986–991. https://doi.org/10.1126/science.1172702.
44. Menon, B., Gullick, N.J., Walter, G.J., Rajasekhar, M., Garrood, T., Evans, H.G., Taams, L.S., and Kirkham, B.W. (2014). Interleukin-17+CD8+ T cells are enriched in the joints of patients with psoriatic arthritis and correlate with disease activity and joint damage progression. Arthritis Rheumatol. *66*, 1272–1281. https://doi.org/10.1002/art.38376.
45. Raychaudhuri, S.K., Abria, C., Mitra, A., and Raychaudhuri, S.P. (2020). Functional significance of MAIT cells in psoriatic arthritis. Cytokine *125*, 154855. https://doi.org/10.1016/j.cyto.2019.154855.
46. Taams, L.S. (2020). Interleukin-17 in rheumatoid arthritis: trials and tribulations. J. Exp. Med. *217*. https://doi.org/10.1084/jem.20192048.
47. Kim, J.H., Han, J.W., Choi, Y.J., Rha, M.S., Koh, J.Y., Kim, K.H., Kim, C.G., Lee, Y.J., Kim, A.R., Park, J., et al. (2020). Functions of human liver CD69(+)CD103(-)CD8(+) T cells depend on HIF-2alpha activity in healthy and pathologic livers. J. Hepatol. *72*, 1170–1181. https://doi.org/10.1016/j.jhep.2020.01.010.
48. Stelma, F., de Niet, A., Sinnige, M.J., van Dort, K.A., van Gisbergen, K., Verheij, J., van Leeuwen, E.M.M., Kootstra, N.A., and Reesink, H.W. (2017). Human intrahepatic CD69 + CD8+ T cells have a tissue resident memory T cell phenotype with reduced cytolytic capacity. Sci. Rep. *7*, 6172. https://doi.org/10.1038/s41598-017-06352-3.
49. Finck, R., Simonds, E.F., Jager, A., Krishnaswamy, S., Sachs, K., Fantl, W., Pe'er, D., Nolan, G.P., and Bendall, S.C. (2013). Normalization of mass cytometry data with bead standards. Cytometry *83*, 483–494. https://doi.org/10.1002/cyto.a.22271.
50. Borcherding, N., Bormann, N.L., and Kraus, G. (2020). scRepertoire: an R-based toolkit for single-cell immune receptor analysis. F1000Res *9*, 47. https://doi.org/10.12688/f1000research.22139.2.
51. Subramanian, A., Tamayo, P., Mootha, V.K., Mukherjee, S., Ebert, B.L., Gillette, M.A., Paulovich, A., Pomeroy, S.L., Golub, T.R., Lander, E.S., and Mesirov, J.P. (2005). Gene set enrichment analysis: a knowledge-based approach for interpreting genome-wide expression profiles. Proc. Natl. Acad. Sci. USA *102*, 15545–15550, papers3:. https://doi.org/10.1073/pnas.0506580102
52. Kolberg, L., Raudvere, U., Kuzmin, I., Vilo, J., and Peterson, H. (2020). gprofiler2 – an R Package for Gene List Functional Enrichment Analysis and Namespace Conversion Toolset g:Profiler. F1000Res *9*. https://doi.org/10.12688/f1000research.24956.2.
53. Raudvere, U., Kolberg, L., Kuzmin, I., Arak, T., Adler, P., Peterson, H., and Vilo, J. (2019). g:Profiler: a web server for functional enrichment analysis

and conversions of gene lists (2019 update). Nucleic Acids Res. *47*, W191–W198. https://doi.org/10.1093/nar/gkz369.

54. Stuart, T., Butler, A., Hoffman, P., Hafemeister, C., Papalexi, E., Mauck, W.M., Hao, Y., Stoeckius, M., Smibert, P., and Satija, R. (2019). Comprehensive integration of single-cell data. Cell *177*, 1888–1902.e1821. https://doi.org/10.1016/j.cell.2019.05.031.

55. Hafemeister, C., and Satija, R. (2019). Normalization and variance stabilization of single-cell RNA-seq data using regularized negative binomial regression. Genome Biol. *20*, 296. https://doi.org/10.1186/s13059-019-1874-1.

56. Choudhary, S., and Satija, R. (2022). Comparison and evaluation of statistical error models for scRNA-seq. Genome Biol. *23*, 27-20. https://doi.org/10.1186/s13059-021-02584-9.

57. Hao, Y., Hao, S., Andersen-Nissen, E., Mauck, W.M., Zheng, S., Butler, A., Lee, M.J., Wilk, A.J., Darby, C., Zager, M., et al. (2021). Integrated analysis of multimodal single-cell data. Cell *184*, 3573–3587.e3529. https://doi.org/10.1016/j.cell.2021.04.048.

58. Becht, E., McInnes, L., Healy, J., Dutertre, C.A., Kwok, I.W.H., Ng, L.G., Ginhoux, F., and Newell, E.W. (2018). Dimensionality reduction for visualizing single-cell data using UMAP. Nat. Biotechnol. https://doi.org/10.1038/nbt.4314.

59. Ramesh, R., Kozhaya, L., McKevitt, K., Djuretic, I.M., Carlson, T.J., Quintero, M.A., McCauley, J.L., Abreu, M.T., Unutmaz, D., and Sundrud, M.S. (2014). Pro-inflammatory human Th17 cells selectively express P-glycoprotein and are refractory to glucocorticoids. J. Exp. Med. *211*, 89–104. https://doi.org/10.1084/jem.20130301.

60. Fergusson, J.R., Smith, K.E., Fleming, V.M., Rajoriya, N., Newell, E.W., Simmons, R., Marchi, E., Björkander, S., Kang, Y.-H., Swadling, L., et al. (2014). CD161 defines a transcriptional and functional phenotype across distinct human T cell lineages. Cell Rep. *9*, 1075–1088. https://doi.org/10.1016/j.celrep.2014.09.045.

STAR★METHODS

KEY RESOURCES TABLE

REAGENT or RESOURCE	SOURCE	IDENTIFIER
Antibodies		
Anti-human CD45 (HI30)-89Y (panel I & II)	Standard Biotools	Cat#3089003B; RRID: AB_2661851
Purified anti-Human CD3 (Maxpar® Ready) Antibody (panel I & II; –116Cd)	BioLegend	Cat#300443; RRID: AB_2562808
Anti-Human CD196/CCR6 (G034E3)-141Pr (panel I)	Standard Biotools	Cat#3141003A; RRID: AB_2687639
Anti-Human CD45RA (HI100)-143ND (panel I & II)	Standard Biotools	Cat#3143006B; RRID: AB_2651156
Anti-Human CD69 (FN50)-144ND (panel I & II)	Standard Biotools	Cat#3144018B; RRID: AB_2687849
Anti-Human CD4 (RPA-T4)-145ND (panel I & II)	Standard Biotools	Cat#3145001B; RRID: AB_2661789
Anti-Human CD8 (RPA-T8)-146ND (panel I & II)	Standard Biotools	Cat#3146001B; RRID: AB_2687641
Purified anti-Human CD107a (LAMP-1) (Maxpar® Ready) Antibody (panel I; –147Sm)	BioLegend	Cat#328635; RRID AB_2563708
Anti-Human S1P1 Monoclonal Antibody (2B9) (panel I; –148ND)	Invitrogen	Cat#MA5-28123; RRID: AB_2745106
Anti-Human CD194/CCR4 (L291H4)-149Sm (panel I)	Standard Biotools	Cat#3149029A
Purified anti-Human CD355 (CRTAM) Antibody (panel I; –150ND)	BioLegend	Cat#339102; RRID: AB_1501238
Anti-Human CD103 (Ber-ACT8)-151Eu (panel I & II)	Standard Biotools	Cat#3151011B; RRID: AB_2756418
Anti-Human TCRgd (11F2)-152Sm (panel I & II)	Standard Biotools	Cat#3152008B; RRID: AB_2687643
Anti-Human TCR Va7.2 (3C10)-153Eu (panel I & II)	Standard Biotools	Cat# 3153024B; RRID: AB_2891190
Purified anti-Human CD294 (CRTH2) Antibody (panel I & II; –154Sm)	BioLegend	Cat#350102; RRID: AB_10639863
Purified anti-mouse/Human Helios Antibody (panel I & II; –155Gd)	BioLegend	Cat#137202; RRID: AB_10900638
Anti-Human CD183/CXCR3 (G025H7)-156Gd (panel I & II)	Standard Biotools	Cat#3156004B; RRID: AB_2687646
Anti-Human CD101 (BB27)-158Gd (panel I)	Standard Biotools	Cat#3158020B
Anti-Human CD197/CCR7 (G043H7)-159Tb (panel I & II)	Standard Biotools	Cat#3159003A; RRID: AB_2714155
Anti-Human CD186/CXCR6 (K041E5)-160Gd (panel I & II)	Standard Biotools	Cat#3160016B
Anti-Human/Mouse Tbet (4B10)-161Dy (panel I & II)	Standard Biotools	Cat#3161014B; RRID: AB_2858233
Anti-Human Foxp3 (PCH101)-162Dy (panel I & II)	Standard Biotools	Cat#3162011A; RRID: AB_2687650
Purified anti-mouse/rat/Human FOXP3 Antibody (150D) (panel I & II; –162Dy)	BioLegend	Cat#320002; RRID: AB_439746
Anti-Human Foxp3 (259D)-162Dy (panel I & II)	Standard Biotools	Cat#3162024A
Anti-Human CD49a/Integrin α1 (TS2/7)-163Dy (panel I)	Standard Biotools	Cat#3163015B; RRID: AB_2893061
Anti-Human CD161 (HP-3G10)-164Dy (panel I & II)	Standard Biotools	Cat#3164009B; RRID: AB_2687651
Anti-Human CD45RO (UCHL1)-165Ho (panel I & II)	Standard Biotools	Cat#3165011B; RRID: AB_2756423
Purified anti-Human Granzyme A Antibody (panel 1; –166Er)	BioLegend	Cat#507202; RRID: AB_315468
Anti-Human/Mouse GATA3 (TWAJ)-167Er (panel I & II)	Standard Biotools	Cat#3167007A; RRID: AB_2927569
Purified anti-Human TCR Vα24-Jα18 (iNKT cell) Antibody (panel I; –168Er & panel II; –171Yb)	Standard Biotools	Cat#342902; RRID: AB_2229301
Anti-Human CD25 (2A3)-169Tm (panel I & II)	Standard Biotools	Cat#3169003B; RRID: AB_2661806
Purified anti-Human CD25 Antibody (panel I & II; –169Tm)	BioLegend	Cat#356102; RRID: AB_2561752
EOMES Monoclonal Antibody (WD1928) (panel I & II; –170Er)	ThermoFisher	Cat#14-4877-82; RRID: AB_2572882
Anti-Human Granzyme B (GB11)-171Yb (panel I)	Standard Biotools	Cat#3171002B; RRID: AB_2687652
Anti-Human Ki-67 (B56)-172Yb (panel I)	Standard Biotools	Cat#3172024B; RRID: AB_2858243

(Continued on next page)

Continued

REAGENT or RESOURCE	SOURCE	IDENTIFIER
Anti-Human CD184/CXCR4 (12G5)-173Yb (panel I)	Standard Biotools	Cat#3173001B
Anti-Human CD279/PD-1 (EH12.2H7)-174Yb (panel I & II)	Standard Biotools	Cat#3174020B; RRID: AB_2868402
Anti-Human Perforin (B-D48)-175Lu (panel I)	Standard Biotools	Cat#3175004B; RRID: AB_2895147
Anti-Human CD127/IL-7Ra (A019D5)-176Yb (panel I & II)	Standard Biotools	Cat#3176004B; RRID: AB_2687863
Purified anti-TCF1 (TCF7) Antibody (panel II; –141Pr)	BioLegend	Cat#655202; RRID: AB_2562103
Anti-Human IL-4 (MP4-25D2)-142ND (panel II)	Standard Biotools	Cat#3142002B
Anti-Human IL-6 (MQ2-13A5)-147Sm (panel II)	Standard Biotools	Cat#3147002B
Anti-Human IL-17A (BL168)-148ND (panel II)	Standard Biotools	Cat#3148008B
Ultra-LEAF™ Purified anti-Human GM-CSF Antibody (panel II; –149Sm)	BioLegend	Cat#502319; RRID: AB_2814393
Anti-Human IL-22 (22URTI)-150ND (panel II)	Standard Biotools	Cat#3150007B; RRID: AB_2810972
Anti-Human IL-2 (MQ1-17H12)-158Gd (panel II)	Standard Biotools	Cat#3158007B; RRID: AB_2864735
Anti-Human TGFbeta (TW4-6H10)-163Dy (panel II)	Standard Biotools	Cat#3163010B
Anti-Human IL-10 (JES3-9D7)-166Er (panel II)	Standard Biotools	Cat#3166008B
Anti-Human IFNg (B27)-168Er (panel II)	Standard Biotools	Cat#3168005B; RRID: AB_2895146
Anti-Human IL-21 (3A3-N2)-172Yb (panel II)	Standard Biotools	Cat#3172011B; RRID: AB_2810975
Purified anti-Human IL-17F (LN2-9C4) Antibody (panel II; –173Yb)	Miltenyi Biotech	Custom
Anti-Human TNFa (Mab11)-175Lu (panel II)	Standard Biotools	Cat#3175023B
TotalSeq™-C0944 anti-Human CD101 (BB27)	BioLegend	Cat#331017; RRID: AB_2832651
TotalSeq™-C0145 anti-Human CD103 (Integrin αE) (Ber-ACT8)	BioLegend	Cat#350233; RRID: AB_2800933
TotalSeq™-C0149 anti-Human CD161 (HP-3G10)	BioLegend	Cat#339947; RRID: AB_2810532
TotalSeq™-C0804 anti-Human CD186/CXCR6 (K041E5)	BioLegend	Cat#356023; RRID: AB_2876677
TotalSeq™-C0143 anti-Human CD196/CCR6 (G034E3)	BioLegend	Cat#353440; RRID AB_2810563
TotalSeq™-C0154 anti-Human CD27 (O323)	BioLegend	Cat#302853; RRID: AB_2800747
TotalSeq™-C0088 anti-Human CD279/PD-1 (EH12.2H7)	BioLegend	Cat#329963; RRID: AB_2800747
TotalSeq™-C0063 anti-Human CD45RA(HI100)	BioLegend	Cat#304163; RRID: AB_2800764
TotalSeq™-C0575 anti-Human CD49a (TS2/7)	BioLegend	Cat#328319; RRID: AB_2832644
TotalSeq™-C0147 anti-Human CD62L (DREG-56)	BioLegend	Cat#304851; RRID:AB_2800770
TotalSeq™-C0146 anti-Human CD69 (FN50)	BioLegend	Cat#310951; RRID: AB_2800810
TotalSeq™-C0179 anti-Human CX3CR1 (K0124E1)	BioLegend	Cat#355705; RRID: AB_2800960
Anti-Human CD3 (UCHT1) PE-Cy7	BioLegend	Cat#300419; RRID: AB_439781
Anti-Human CD4 (SK3) PerCP-Cy5.5	BioLegend	Cat#344607; RRID: AB_1953236
Anti-Human CD8 (HIT8a) FITC	BioLegend	Cat#344703; RRID: AB_314110
Anti-Human CD14 (REA599) APCVio770	Miltenyi Biotech	Cat#130-110-522; RRID: AB_2655063
**Chemicals, Peptides, and Recombinant Proteins**		
DAPI DNA stain	Invitrogen	Cat#D1306
EQ Element Calibration Beads	Fluidigm	Cat#201078
Foxp3/Transcription Factor Staining Buffer Set	eBioscience	Cat#00-5523-00
GolgiStop™ Protein Transport Inhibitor	BD Biosciences	CAT#554724
Ionomycin	Sigma-Aldrich	CAT#I9657
Iridium (Ir) nucleic acid intercalator	Fluidigm	Cat#201192B
Maxpar® Cell Acquisition Solution (CAS)	Fluidigm	Cat#201244
Phorbol 12-myristate 13-acetate (PMA)	Sigma-Aldrich	CAT#P8139
Rhodium (103Rh)-intercalator	Fluidigm	Cat#201103A
TruStain FcX™ (Fc Receptor Blocking Solution)	BioLegend	Cat#422301

(*Continued on next page*)

Continued

REAGENT or RESOURCE	SOURCE	IDENTIFIER
Critical Commercial Assays		
Dead Cell Removal Kit	Miltenyi Biotec	Cat#130-090-101
REAlease® CD3 MicroBead Kit, Human	Miltenyi Biotec	Cat#130-117-038
Chromium Next GEM Single Cell 5′ Kit v2	10x Genomics	Cat#1000263
Chromium Next GEM Chip K Single Cell Kit	10x Genomics	Cat#1000287
Chromium Single Cell Human TCR Amplification Kit	10x Genomics	Cat#1000252
Dual Index Kit TT Set A	10x Genomics	Cat#1000215
Dual Index Kit TN Set A	10x Genomics	Cat#1000250
Library Construction Kit	10x Genomics	Cat#1000190
5′ Feature Barcode Kit	10x Genomics	Cat#1000256
Deposited Data		
scRNAseq data files	GEO	GSE216914
Software and Algorithms		
NormalizerR normalization software	Finck et al.[49]	N/A
CATALYST pipeline	Nowicka et al.[24]	N/A
CellRanger v6.1.1	10x Genomics	N/A
Seurat 4.1.1	Satija Lab	N/A
scRepertoire 1.7.2	Borcherding et al.[50]	N/A
GSEA version 4.2.3	Subramanian et al.[51]	N/A
gprofiler2	Kolberg et al.[52]; Raudvere et al.[53]	N/A

RESOURCE AVAILABILITY

Lead contact

Further information and requests for resources and reagents should be directed to and will be fulfilled by the lead contact, Professor Leonie Taams (she/her) (leonie.taams@kcl.ac.uk).

Materials availability

This study did not generate new unique reagents.

Data and code availability

- According to UK research councils' Common Principles on Data Policy, all single-cell RNA-seq data have been deposited at GEO under accession number GSE216914 and are publicly available as of the date of publication.
- This paper does not report original code. Publicly available code was adapted and/or combined to meet the specification of our dataset analysis, as indicated in STAR Methods for CyTOF and scRNAseq analyses.
- Any additional information required to reanalyze the data reported in this paper is available from the lead contact upon request.

EXPERIMENTAL MODEL AND SUBJECT DETAILS

Participant recruitment and ethical approval

Synovial fluid (SF) samples were obtained from 8 patients with Psoriatic Arthritis (PsA; 4 males and 4 females, 25 to 71 years old) and 5 Rheumatoid Arthritis (RA; 1 male and 4 females, 41 to 74 years old) recruited from Guy's Hospital Rheumatology Department, with written informed consent from all participants. The study was approved by the Bromley Research Ethics Committee (06/Q0705/20) and Harrow Research Ethics Committee (17/LO/1940). Patient demographics and clinical information for samples included for CyTOF and scRNAseq is available in Table 1.

METHOD DETAILS

Cell isolation

SF mononuclear cells (SFMC) were isolated from synovial fluid by conventional density gradient centrifugation using Lymphoprep™ (Axis-Shield, Oslo, Norway). Cells were cryopreserved in culture medium containing 90% FCS (Gibco, Invitrogen, UK) and 10% DMSO (Fisher Scientific, Loughborough, UK) in liquid nitrogen until use.

CyTOF staining and sample acquisition

Upon thawing, dead cells were removed using the Dead Cell Removal kit and CD3+ T cells were isolated using the REAlease CD3 Microbead kit (both kits Miltenyi Biotec, Bergisch Gladbach, Germany) according to manufacturer's guidelines. Purified CD3+ T-cells (3×10^6) were left unstimulated or were stimulated for 3 h with PMA (50 ng/mL), ionomycin (750 ng/mL, (Sigma-Aldrich, St. Louis, Missouri, United States) in the presence of GolgiStop (BD Biosciences, Franklin Lakes, New Jersey) to assess intracellular cytokine expression. Wash steps were performed at 1,200rpm for 10 min at room temperature (RT) unless otherwise stated. Details for both CyTOF antibody panels are listed in key resources table (panel I for samples without PMA/ionomycin stimulation; panel II for samples stimulated with PMA/ionomycin). After 3 h, cells were washed with PBS then stained with 1X rhodium (Rh)-intercalator (Fluidigm) in PBS for 15 min at RT for viability assessment. Next, cells were washed twice with cell staining medium (CSM, 0.5% BSA +0.02% NaN3 in PBS) before stained with extracellular antibodies for 30 min at RT. Cells were washed twice with CSM then fixed and permeabilised using the FoxP3/Transcription Factor Staining Buffer set (eBioscience, UK) according to manufacturer's instructions. After washing with permeabilization buffer, cells were stained with intracellular and intranuclear markers for 30 min at 4°C (panel I unstimulated cells; panel II stimulated cells). Cells were washed with permeabilization buffer then stored overnight at 4°C resuspended in 125nM iridium (Ir)-intercalator in 4% paraformaldehyde (PFA). Samples were washed and resuspended in Maxpar CAS buffer (Fluidigm) with EQ Element Calibration Beads (Fluidigm) and acquired on a Helios mass cytometer (Fluidigm).

Cell sorting for single cell RNA sequencing

Cryopreserved SFMCs from four patients with PsA were thawed and 2×10^6 SFMCs were stained for 30 min at 4°C with a combination of fluorescently labeled antibodies and Cellular Indexing of Transcriptomes and Epitopes by sequencing (CITE-Seq) antibodies in the presence of Fc receptor blocker. DAPI was added to samples immediately prior to sorting and live synovial CD8+ T cells were then FACS sorted using a FACSAria/LSRII and retained on ice ahead of library preparation.

Single cell library preparation and sequencing

20,000 FACS sorted synovial CD8+ T cells per patient were loaded for gene expression and immune profiling using the 10X Genomics Chromium Single Cell platform. Single cell libraries were created using the Chromium Single-Cell 5′ Reagent Kits v2. Libraries were sequenced on the NextSeq 2000 platform.

QUANTIFICATION AND STATISTICAL ANALYSIS

CyTOF data analysis

EQ Element Calibration Beads were used for signal normalization across sample FCS files using the MATLAB version of the NormalizerR normalization software.[49] Live CD8+ and CD4+ T cells were gated and exported independently for downstream analysis using FlowJo software (v10). Data were analyzed using a customised version of the CATALYST pipeline.[24] Uniform manifold approximation and projection (UMAP)s were generated based on the arcsinh-transformed expression of markers evaluated on the cells; the lowest cell number among the different donors was used as common threshold for UMAP generation. Populations were identified with FlowSOM after the metaclustering step with ConsensusClusterPlus with no downsampling. For quantification of contextual feature enrichment of the clusters identified by FlowSOM and ConsensusClusterPlus, marker enrichment modeling (MEM) was performed.[25] For differential analysis of population abundances, a generalized linear mixed model (GLMM) within the CATALYST analysis pipeline was used. Additional statistical testing for CyTOF data was performed with GraphPad Prism v9.0 (GraphPad, San Diego, CA, USA) using the multiple Mann-Whitney test as indicated in figure legends.

Pre-processing of scRNAseq data

Raw reads were aligned to the human transcriptome and "multi" option of CellRanger software package (v6.1.1) with default parameters. Outputs from CellRanger were loaded into Seurat (v4.0.4). Cells with <500 or >3,000 genes, <500 or >15,000 UMIs, >5,000 ADT UMIs, expression of non-T cell markers (CD14+/CD19+) or >10% mitochondrial genes were filtered out. In addition, for cells where TCR sequencing data were available, multiplets were excluded by removal of cells expressing more than one TCRβ chain or more than two TCRα chains.

scRNAseq analysis

scRNAseq data analysis was performed using Seurat. After filtering, the four datasets were individually normalised using the SCTransform function (v2) with regression of the percentage of mitochondrial genes (RNA assays) and the NormalizeData and ScaleData functions (ADT assays) and then integrated.[54–56] Dimensionality reduction was performed separately for the integrated SCT and integrated ADT assays with exclusion of TCR genes from the variable gene list. Weighted Nearest Neighbor (WNN) analysis[57] was used to integrate RNA and protein expression, using 30 dimensions and 11 dimensions respectively, to construct a WNN graph. Clustering was then performed on the WNN of the integrated SCT and ADT assays using the FindClusters command with resolution 0.7 and visualised using UMAPs.[58] Wilcoxon rank sum tests were performed to find differentially expressed genes in each cluster using the FindMarkers and FindAllMarkers functions in Seurat for scRNA-seq. Adjusted p-values <0.05 were considered statistically significant.

GSEA was performed using GSEA v4.2.3[51]. Functional enrichment analysis of differential gene lists to identify significantly enriched biological functions and pathways was performed using the gost function in gprofiler2[52,53] using standard settings. Lung and spleen CD8+ T_{RM} signature for GSEA was sourced from.[15] Gene sets associated with human Th17 and CD8+CD161+ and MAIT cell signatures for gost function in gprofiler2 were obtained from[59,60] respectively. Gene signature of *in vitro* TGFβ stimulated human CD8+ T cells was sourced from.[29]

Single cell TCR sequencing analysis

Diversity, Morisita and clone-sharing analysis was performed for clusters with >100 TCR sequences, for each donor, using the scRepertoire package in R[60].

Cell Reports, Volume *42*

Supplemental information

Psoriatic and rheumatoid arthritis joints differ in the composition of CD8+ tissue-resident memory T cell subsets

Giovanni A.M. Povoleri, Lucy E. Durham, Elizabeth H. Gray, Sylvine Lalnunhlimi, Shichina Kannambath, Michael J. Pitcher, Pawan Dhami, Thomas Leeuw, Sarah E. Ryan, Kathryn J.A. Steel, Bruce W. Kirkham, and Leonie S. Taams

Figure S1

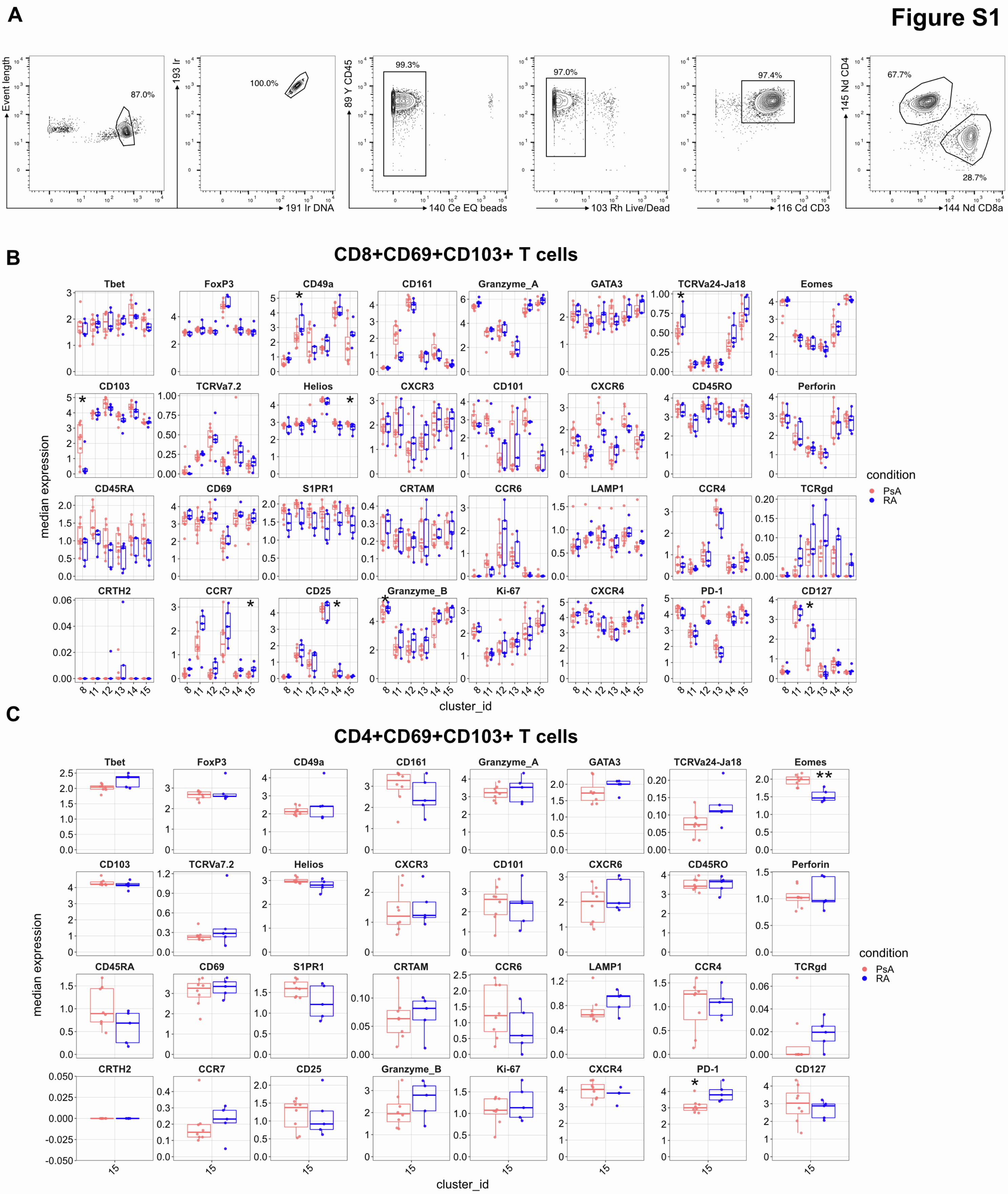

Figure S1. Gating strategy and phenotypic comparison of synovial CD8+CD69+CD103+ and CD4+CD69+ CD103+ TRM cells clusters in patients with PsA and RA; Related to Figures 1,2. (A) Representative plots demonstrating the gating strategy for sample cleanup and export of total CD8+ and CD4+ T-cells from patients with PsA or RA pre CyTOF analysis inputted into the modified CATALYST analysis pipeline. The gating strategy is shown for intact cells (191Ir+ DNA stain), cells (191Ir+193Ir+), no beads (89Y CD45+140Ce EQ beads-), live (89Y CD45+ 103Rh-), CD3+ (89Y CD45+ 116Cd CD3+) and CD8+ (144Nd CD8a+ 145Nd CD4-) or CD4+ (144Nd CD8a- 145Nd CD4+) cells. (B,C) Cumulative data showing the median arcsinh-transformed expression of the markers across the identified CD8+CD69+CD103+ (B) or CD4+CD69+CD103+ (C) TRM cell clusters from Figure 1C. Values for the two disease conditions are indicated as PsA in red and RA in blue. Boxplots show median and IQR. Data analysed by multiple Mann-Whitney tests with *p<0.05. Data from patients with PsA (n=8) or RA (n=5) are shown.

Figure S2

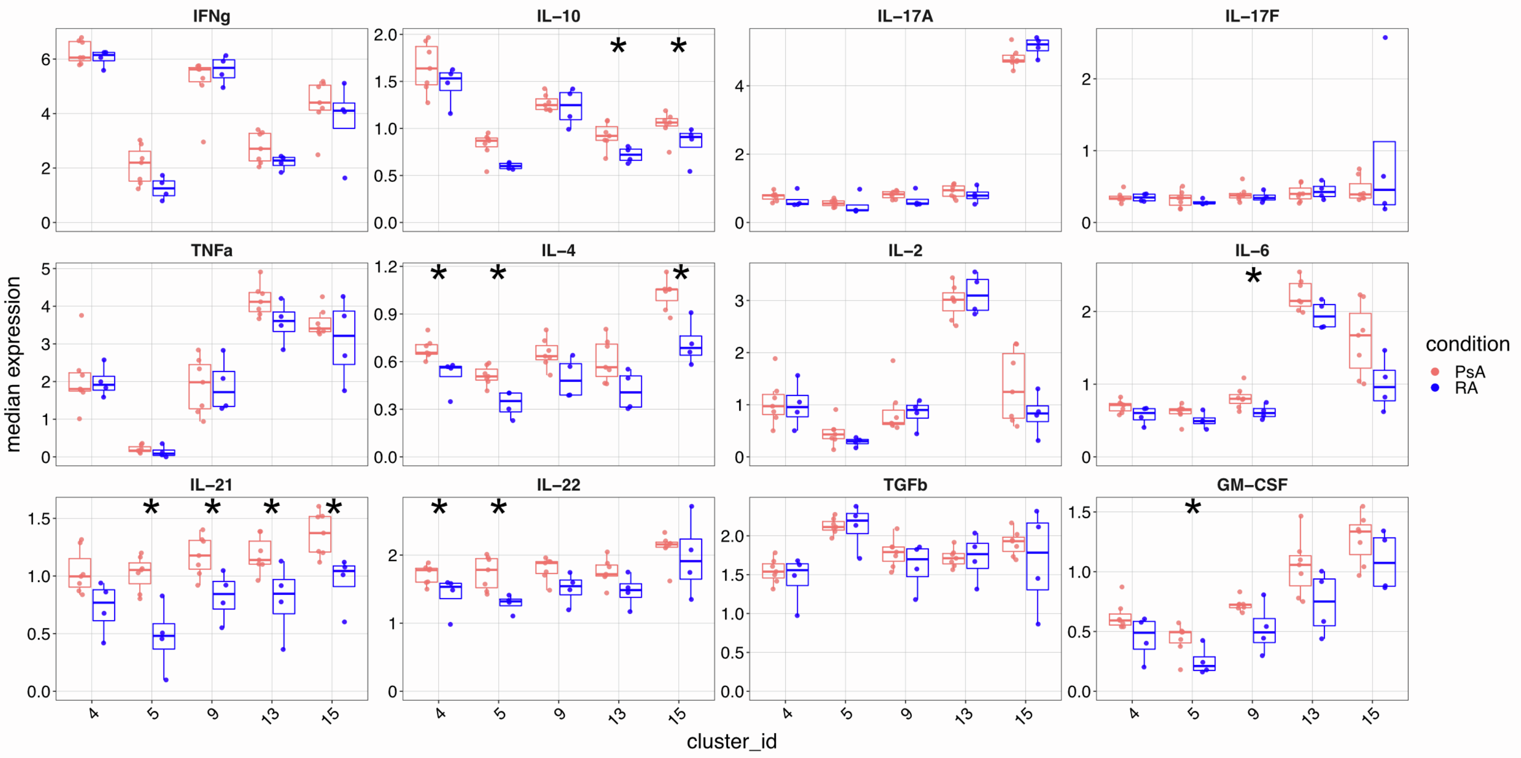

Figure S2. Cytokine expression comparison of synovial CD8+CD69+CD103+ TRM cells clusters in patients with PsA and RA; Related to Figure 3. Cumulative data showing the arcsinh-transformed, median expression of cytokines across the identified CD8+CD69+CD103+ TRM cell clusters from Figure 3B (PsA in red and RA in blue). Boxplots show median and IQR. Data analysed by multiple Mann-Whitney tests and *p<0.05. Data from patients with PsA (n=7) or RA (n=4) are shown.

Figure S3

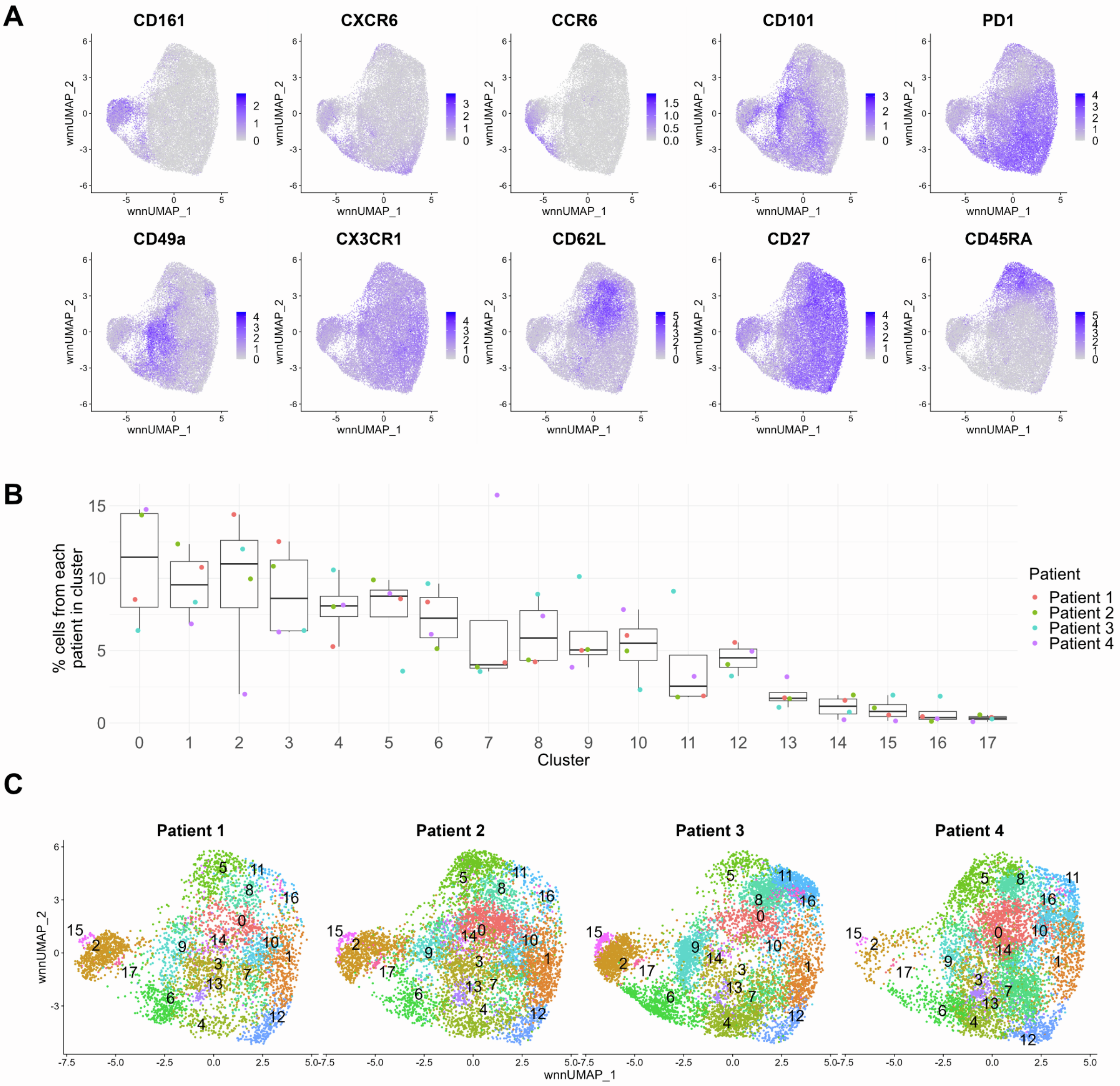

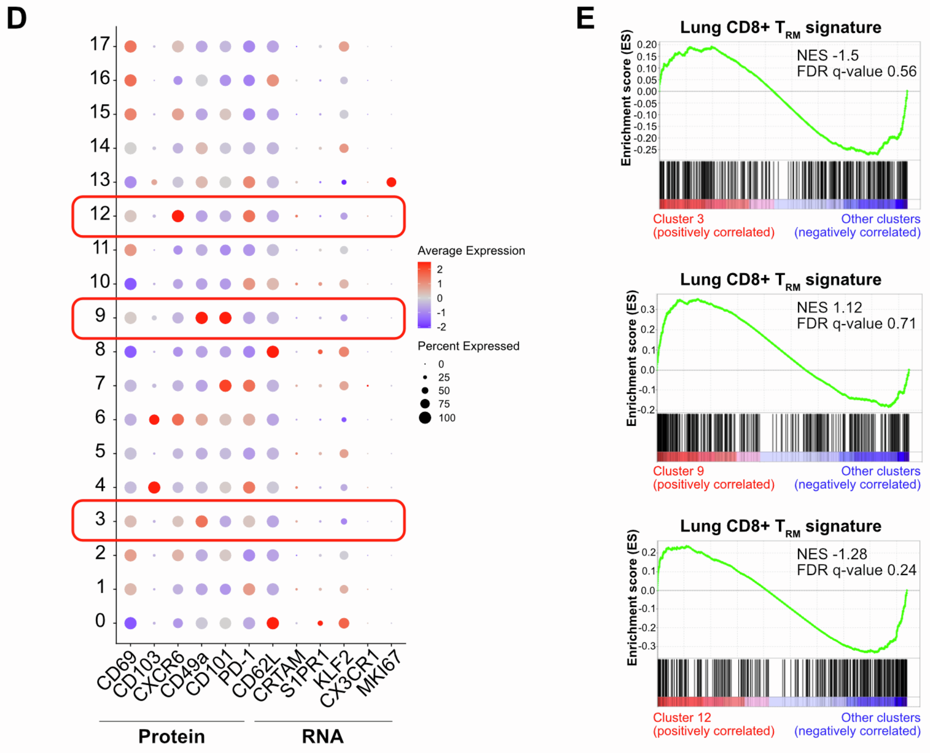

Figure S3. scRNAseq clusters representation among donors and evaluation of synovial CD8+CD69+ CD103- clusters in patients with PsA for TRM cell gene signature; Related to Figure 4. (A) UMAPs showing cell protein expression levels of indicated proteins in CD8+ T-cells from patients with PsA (n=4). (B) Boxplot showing distribution of cells from each patient across clusters (n=4). (C) Individual UMAP for each patient showing cells coloured according to the 18 populations obtained after Seurat clustering. (D) Dot plot heatmap showing the average expression (colour) and percentage of cells (dot size) expressing the indicated genes or proteins associated with a TRM cell phenotype (n=4). (E) GSEA plots for lung CD8+ TRM signature, comparing clusters 3, 9 and 12 with all other clusters; n = 4 per group. Normalized enrichment score (NES) and multiple-test adjusted q-value from GSEA are shown.

Figure S4

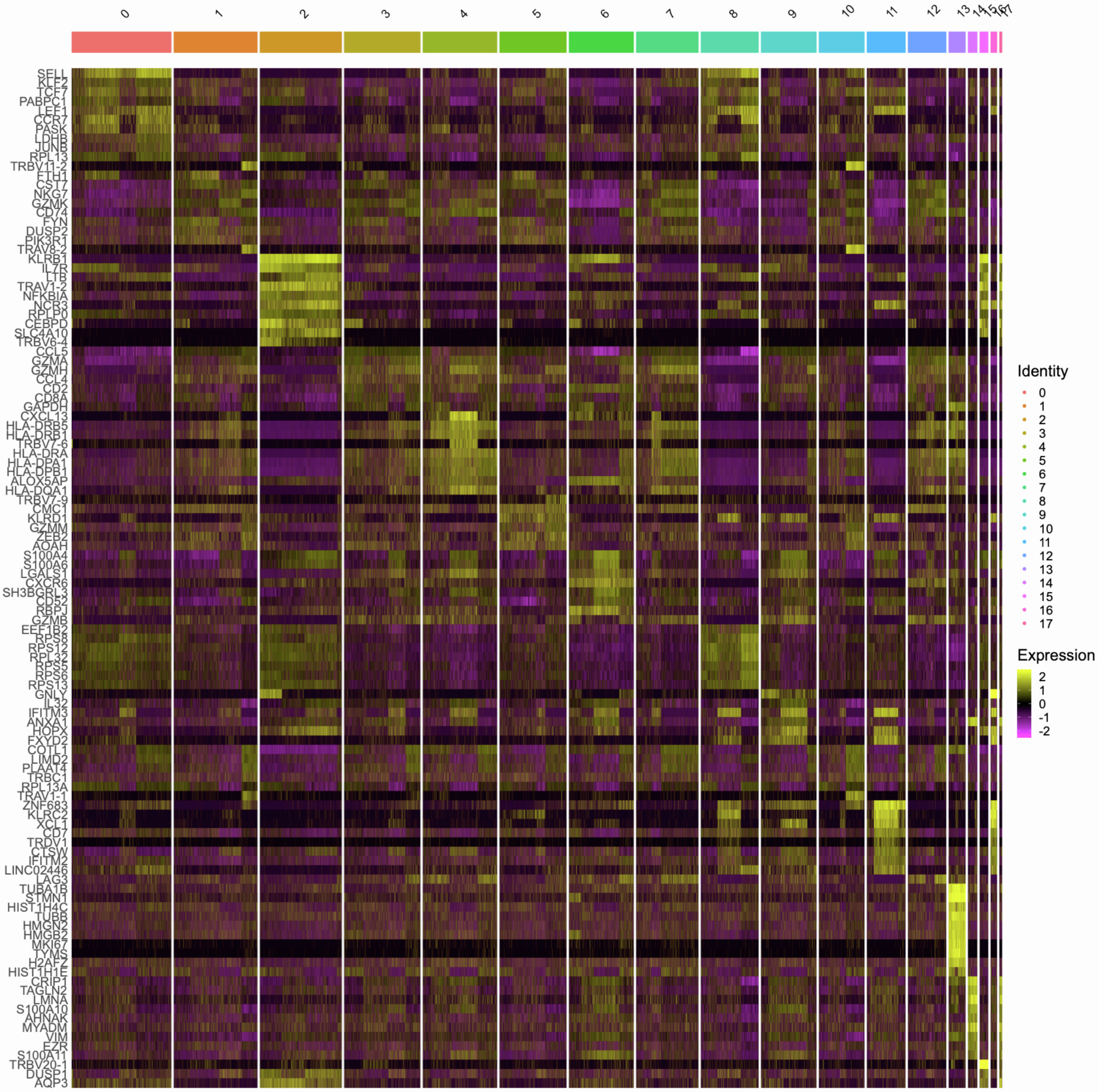

Figure S4. Heatmap of top 10 differentially expressed genes from each cluster identified in synovial CD8+ cells from patients with PsA; Related to Figure 4. Heatmap showing the top 10 upregulated genes for each of the 18 clusters from the integrated analysis of CD8+ T cells from n= 4 patients. Upregulated genes were identified by Wilcoxon rank sum test. Within each cluster, genes are ordered by highest to lowest LogFC of SCTransformed RNA expression. Adjusted P-values < 0.05 were considered statistically significant.

Figure S5

A

Cluster 13

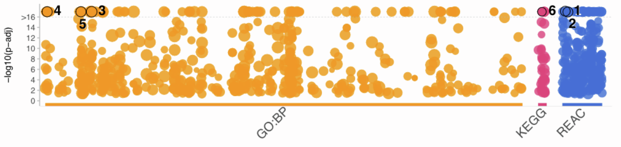

id	term_id	term_name	q_value
1	REAC:R-HSA-1640170	Cell Cycle	1.13E-131
2	REAC:R-HSA-69278	Cell Cycle, Mitotic	6.67E-114
3	GO:0007049	cell cycle	1.16E-114
4	GO:0000278	mitotic cell cycle	1.58E-96
5	GO:0006259	DNA metabolic process	2.98E-72
6	KEGG:04110	Cell cycle	2.83E-25

B

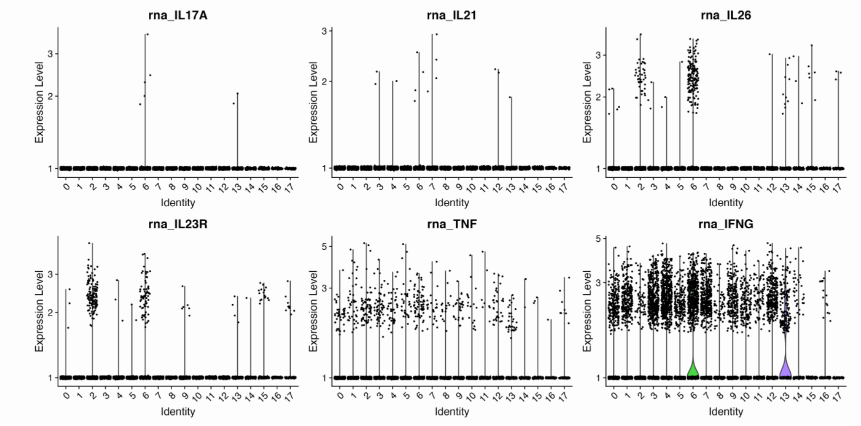

C

Cluster 4

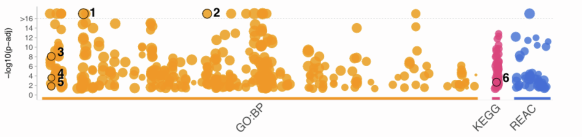

id	term_id	term_name	q_value
1	GO:0006955	immune response	1.10E-31
2	GO:0042110	T cell activation	5.50E-23
3	GO:0001909	leukocyte mediated cytotoxicity	8.54E-09
4	GO:0001913	T cell mediated cytotoxicity	2.6E-04
5	GO:0001910	regulation of leukocyte mediated cytotoxicity	1.5E-02
6	KEGG:04650	Natural killer cell mediated cytotoxicity	2.2E-03

Cluster 6

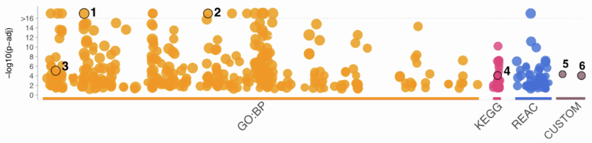

id	term_id	term_name	q_value
1	GO:0006955	immune response	7.19E-19
2	GO:0042110	T cell activation	2.80E-21
3	GO:0002250	adaptive immune response	8.32E-06
4	KEGG:04659	Th17 cell differentiation	8.50E-05
5	Ramesh et al. 2014	Human Th17 signature	2.24E-04
6	Fergusson et al. 2014	CD8+CD161+ and MAIT cells	2.86E-04

D

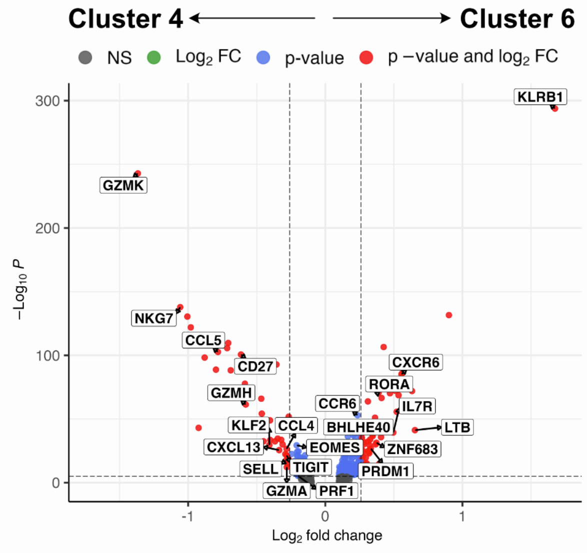

E

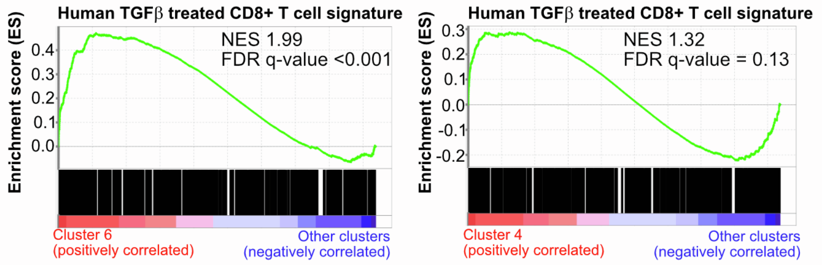

Figure S5. Functional enrichment analysis and gene signature differences between synovial CD8+CD69+CD103+ TRM clusters identified in patients with PsA; Related to Figure 4. (A) Manhattan plot showing functional enrichment analysis of differentially upregulated genes in cluster 13 compared to all other clusters. The x-axis shows the biological functions and pathways data sources (terms) and y-axis shows the enrichment p-values (−log10). Each circle corresponds to a single term from an annotation source (colour) and shows the total number of genes annotated to the corresponding term (circle size). Chosen terms for each cluster are indicated with numbers on the plot and corresponding id, name and statistics are shown in the table below the Manhattan plot. (B) Violin plots showing gene expression levels of type 17 and inflammatory cytokines in the different Seurat clusters identified from SF CD8+ T-cells from patients with PsA (n=4). (C) Manhattan plots, with associated terms and statistics, showing functional enrichment analysis of differentially upregulated genes in clusters 4 (top) and 6 (bottom) compared to all other clusters. (D) Volcano plot showing significant genes differentially expressed in cluster 6 compared to cluster 4. Differentially expressed genes are identified in blue (q-value <0.05) or red (q-value <0.05 with a ±1.2-fold change (n=4 patients). (E) GSEA plots for human TGFβ treated CD8+ T-cell signature, comparing Cluster 6 (left) and 4 (right) with all other clusters; n = 4 per group. Normalized enrichment score (NES) and multiple-test adjusted q-value from GSEA are shown.

Figure S6

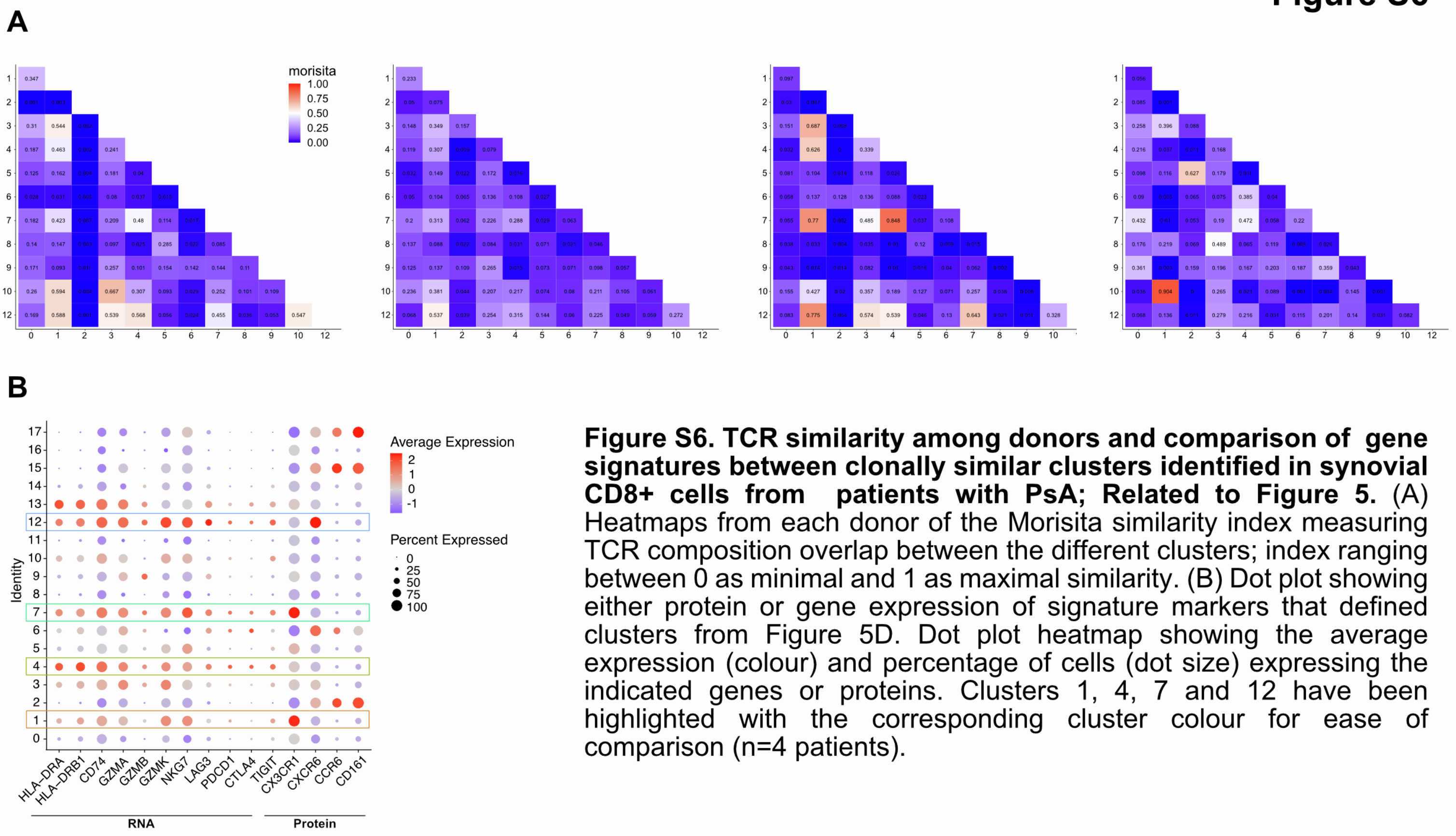

Figure S6. TCR similarity among donors and comparison of gene signatures between clonally similar clusters identified in synovial CD8+ cells from patients with PsA; Related to Figure 5. (A) Heatmaps from each donor of the Morisita similarity index measuring TCR composition overlap between the different clusters; index ranging between 0 as minimal and 1 as maximal similarity. (B) Dot plot showing either protein or gene expression of signature markers that defined clusters from Figure 5D. Dot plot heatmap showing the average expression (colour) and percentage of cells (dot size) expressing the indicated genes or proteins. Clusters 1, 4, 7 and 12 have been highlighted with the corresponding cluster colour for ease of comparison (n=4 patients).

Figure S7

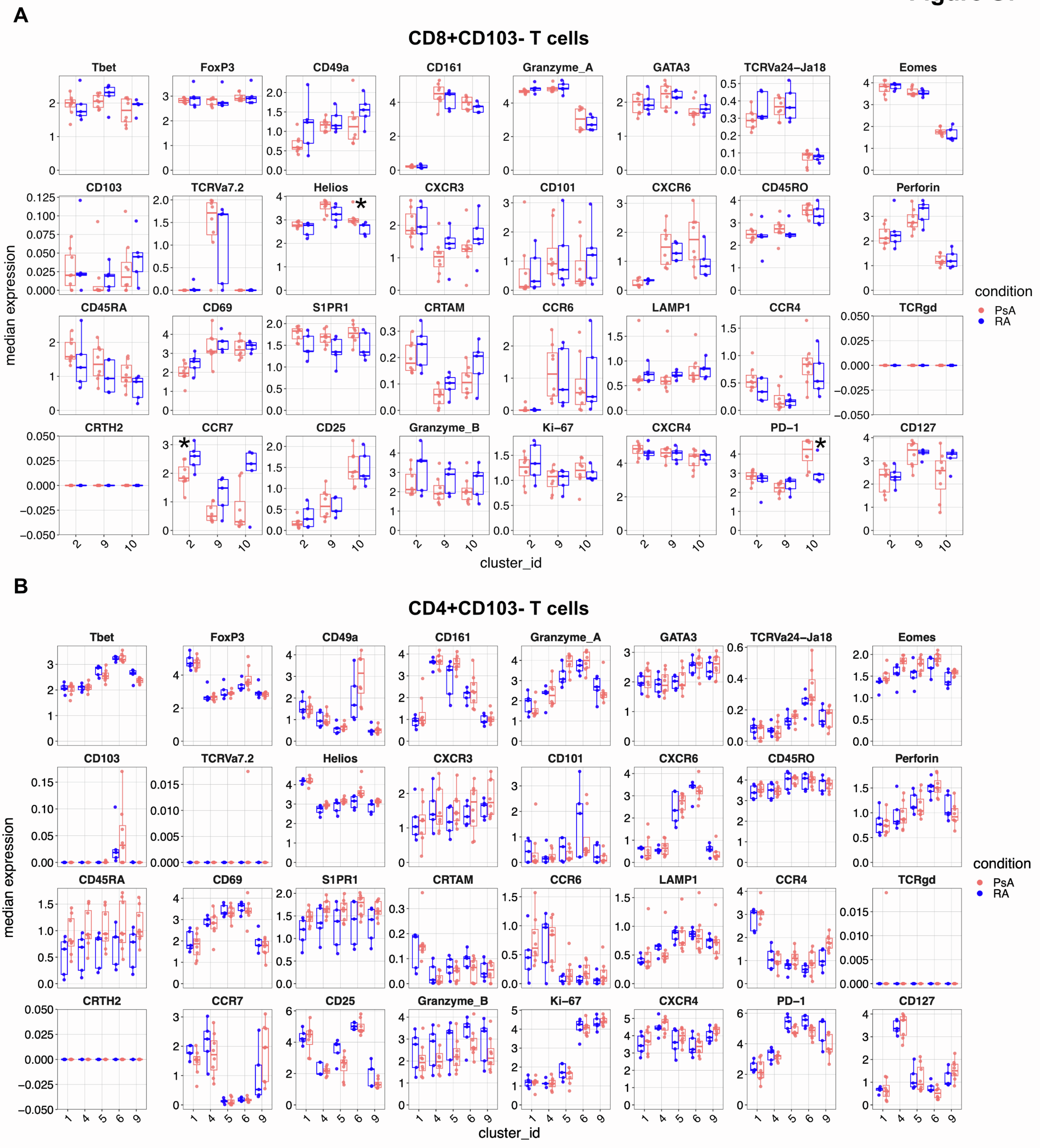

Figure S7. Phenotypic comparison of synovial CD8+CD103- and CD4+CD103- clusters in patients with PsA and RA; Related to Figure 6. Cumulative data showing the median arcsinh-transformed expression of the markers across the significantly enriched CD8+CD103- (A) or CD4+ CD103- cell clusters in either PsA (red) or RA (blue) from Figure 6. Boxplots show median and IQR. Data analysed by multiple Mann-Whitney tests and *$p<0.05$. Data from patients with PsA (n=8) or RA (n=5) are shown.
